# PARP1 protects from benzo[a]pyrene diol epoxide-induced replication stress and mutagenicity

**DOI:** 10.1007/s00204-017-2115-6

**Published:** 2017-12-01

**Authors:** Jan M. F. Fischer, Tabea Zubel, Kirsten Jander, Jelena Fix, Irmela R. E. A. Trussina, Daniel Gebhard, Jörg Bergemann, Alexander Bürkle, Aswin Mangerich

**Affiliations:** 1 Molecular Toxicology Group, Department of Biology, University of Konstanz, 78457 Konstanz, Germany; 2 Konstanz Research School Chemical Biology, University of Konstanz, Konstanz, Germany; 3 Department of Life Sciences, Albstadt-Sigmaringen University of Applied Sciences, Albstadt, Germany

**Keywords:** PARP 1, Poly(ADP-ribosyl)ation, Benzo[a]pyrene, BPDE, Replication stress

## Abstract

Poly(ADP-ribosyl)ation (PARylation) is a complex and reversible posttranslational modification catalyzed by poly(ADP-ribose)polymerases (PARPs), which orchestrates protein function and subcellular localization. The function of PARP1 in genotoxic stress response upon induction of oxidative DNA lesions and strand breaks is firmly established, but its role in the response to chemical-induced, bulky DNA adducts is understood incompletely. To address the role of PARP1 in the response to bulky DNA adducts, we treated human cancer cells with benzo[a]pyrene 7,8-dihydrodiol-9,10-epoxide (BPDE), which represents the active metabolite of the environmental carcinogen benzo[a]pyrene [B(a)P], in nanomolar to low micromolar concentrations. Using a highly sensitive LC-MS/MS method, we revealed that BPDE induces cellular PAR formation in a time- and dose-dependent manner. Consistently, PARP1 activity significantly contributed to BPDE-induced genotoxic stress response. On one hand, PARP1 ablation rescued BPDE-induced NAD^+^ depletion and protected cells from BPDE-induced short-term toxicity. On the other hand, strong sensitization effects of PARP inhibition and PARP1 ablation were observed in long-term clonogenic survival assays. Furthermore, PARP1 ablation significantly affected BPDE-induced S- and G2-phase transitions. Together, these results point towards unresolved BPDE-DNA lesions triggering replicative stress. In line with this, BPDE exposure resulted in enhanced formation and persistence of DNA double-strand breaks in PARP1-deficient cells as evaluated by microscopic co-localization studies of 53BP1 and γH2A.X foci. Consistently, an *HPRT* mutation assay revealed that PARP inhibition potentiated the mutagenicity of BPDE. In conclusion, this study demonstrates a profound role of PARylation in BPDE-induced genotoxic stress response with significant functional consequences and potential relevance with regard to B[a]P-induced cancer risks.

**Electronic supplementary material:**

The online version of this article (10.1007/s00204-017-2115-6) contains supplementary material, which is available to authorized users.

## Introduction

Thousands of DNA lesions occur in every cell during a day by the encounter with genotoxic agents form endogenous and exogenous sources. As a result, various DNA repair mechanisms have evolved during evolution to ensure genomic integrity (Ciccia and Elledge 2010). One of those mechanisms is nucleotide excision repair (NER)—a versatile molecular machinery involved in the removal of bulky and helix distorting DNA adducts (Marteijn et al. 2014). NER is unique among DNA repair mechanisms, since it detects a wide spectrum of DNA lesions only with a small set of common damage recognition and repair initiators. The underlying feature of all these structurally different DNA lesions is the varying degree of DNA kinking and helix distortion. The efficiency of lesion removal is mainly determined by the degree of distortion and thus the initial binding and verification of the lesion site (Marteijn et al. 2014). NER is a multistep process neatly choreographed by the sequential assembly of almost 30 proteins. The single steps involve initial damage recognition, local DNA unwinding and damage verification, dual incision on the damaged strand and removal of the lesion-containing oligonucleotide, re-synthesis of DNA, and sealing of the nick (Scharer 2013). Two sub-pathways can initiate the NER machinery, the global-genome NER (GG-NER), responsible for maintenance of the whole genome, and the transcription-coupled NER (TC-NER), involved in the detection and removal of lesion sites in actively transcribed genes (Spivak 2015). Among the most common NER, lesions are UV-light-induced 6-4PPs’ and CPDs’ lesions, but also bulky DNA lesions caused by chemicals such as polycyclic aromatic hydrocarbons (PAH).

PAHs are a class of several hundred chemical compounds, many of which are known to be persistent environmental toxins, mutagens, and carcinogens (EPA 2006; Kim et al. 2013). One of those, benzo[a]pyrene (B[a]P), arises from incomplete combustion of organic matter and can be ubiquitously found in automobile exhaust, industrial emission, cigarette smoke, and charcoal-broiled meat. It has been detected in food and plants (0.1–150 µg/kg), drinking water (2.5–9 ng/l), and air (1.3–500 ng/m^3^) (Angerer et al. 1997; Madureira et al. 2014). B[a]P is a pro-carcinogen and not reactive per se. However, upon entering the cell, it binds the aryl hydrocarbon receptor (AHR) and triggers the expression of the biotransforming CYP450 enzymes involved in xenobiotic metabolism. As a result, CYP1A1, CYP1B1, and the epoxide hydrolase (mEH) convert B[a]P to the highly reactive, cytotoxic agent benzo[a]pyrene-7,8-dihydrodiol-9,10-epoxide (BPDE), which reacts with DNA mainly at the *N* ^*2*^ position of guanine (Moserova et al. 2009). Doses of 0.01–0.1-µM BPDE form 800–9600 bulky DNA adducts, which can be detected and repaired by the NER pathway (Akerman et al. 2004; Gelboin 1980; Kim et al. 1998). However, if not repaired, BPDE-DNA adducts are the major cause for BPDE’s toxicity, resulting in replicative stress and genomic instability. Treatment of cells with BPDE induces apoptosis via p53, BAX and JNK as well as necrosis, caused by NAD^+^ depletion due to PARP1 overactivation (Donauer et al. 2012; Lin and Yang 2008; Wani et al. 2000). Furthermore, BPDE is highly mutagenic, potentially leading to tumorigenic transformation (Akerman et al. 2004; Deng et al. 2014; Dreij et al. 2005; Lin and Yang 2008; Pavanello et al. 2008).

PARP1 is involved in a broad spectrum of cellular processes, many of which are associated with genome maintenance (Ray Chaudhuri and Nussenzweig 2017). It has been reported to interact in particular with DNA single and double-strand breaks, however, also other substrates, such as UV-induced DNA damage, DNA hairpins and cruciform DNA function as PARP1 substrates (Lonskaya et al. 2005; Purohit et al. 2016). In response to binding to different DNA structures, several modes of PARP1 activation are conceivable, probably resulting in varying degrees of catalytic activity. Thus, the magnitude of PARP1 activity depends on the type of DNA damage (e.g., blunt end vs. base overhang) (Benjamin and Gill 1980; D’Silva et al. 1999; Pion et al. 2005). In any case, upon activation, PARP1 uses NAD^+^ as a substrate to covalently attach an ADP-ribose unit to itself (i.e., automodification) or other target proteins under the release of nicotinamide as a by-product. Subsequently, this mono(ADP-ribose) unit can be further elongated to form polymer chains of up to 200 moieties (Hottiger 2015; Ueda and Hayaishi 1985). PARP1 facilitates the repair of DNA lesions by a wide array of functions. For example, PARylation locally opens the chromatin and forms a platform to facilitate the recruitment and assembly of DNA repair factors, organizes access and removal of repair factors, and influences their enzymatic activities (Fischer et al. 2014; Posavec Marjanovic et al. 2016; Ray Chaudhuri and Nussenzweig 2017).

While the role of PARP1 in DNA strand break and base excision repair is well characterized, the understanding of its functions in response to bulky DNA lesions is only emerging. Recent studies suggested that PARP1 is an important factor for an efficient NER process and facilitates the removal of UV photoproducts (Fischer et al. 2014; Pines et al. 2012; Robu et al. 2013, 2017). PARP1 has been shown to physically interact with several factors of the NER machinery, to covalently or non-covalently modify them with PAR, and thus alter their functionality and subcellular localization. Thus, CSB interacts with PARP1 and PAR, and its ATPase activity was reported to be inhibited upon this interaction (Scheibye-Knudsen et al. 2014; Thorslund et al. 2005). XPC is modified with PAR in a covalent and non-covalent manner and is recruited to damage sites in a PARP1- and PAR-dependent manner (Robu et al. 2013, 2017). XPA has been shown to interact with PARP1 and PAR, and this interaction functions as a reciprocal regulatory mechanism between the NER pathway and PARP1. Thus, XPA stimulates PARP1’s catalytic activity, whereas PARylation regulates XPA’s DNA-binding ability (Fischer et al. 2014; King et al. 2012). Furthermore, DDB2 has been shown to stimulate PARP1 activity in the presence of UV photoproducts, resulting in chromatin decondensation and recruitment of the chromatin remodeler ALC1. PARylation of DDB2 stabilizes the protein, preventing ubiquitination and proteasomal degradation (Luijsterburg et al. 2012; Pines et al. 2012).

The previous studies concerning the role of PARylation in the response to bulky DNA lesions mainly focused on its role in the response to UV-light-induced photoproducts. However, not all NER substrates are processed in the same manner and remarkable variations exist in the degree of damage recognition, efficiency and pace of lesion removal, as well as composition of essential NER factors (Lee et al. 2014; Marteijn et al. 2014; Robu et al. 2013; Wood 1999). Since the role of PARP1 in the response to chemical-induced, bulky DNA adducts is largely uncharacterized, we addressed this question using the B[a]P metabolite BPDE. While our analyses did not identify a role for PARP1 in BPDE-induced NER mechanisms, we revealed a major protective role for PARP1 in BPDE-induced replication stress with significant functional consequences in terms of BPDE-induced cytotoxicity and mutagenicity.

## Materials and methods

### Cell culture

CHO, HeLa Wt, and HeLa *PARP1* knock-out cells (clones KO1 and KO2) (Rank et al. 2016) were cultured in DMEM (Thermo Fisher Scientific) supplemented with 10% FCS, 100-U/ml streptomycin, and 100-µg/ml penicillin (termed ‘complete growth medium’). Primary human foreskin fibroblasts were cultured in DMEM (Thermo Fisher Scientific), supplemented with 10% FCS and 50-µg/ml gentamycin. Cells were kept at 37 °C, 95% humidity, and 5% CO_2_.

### BPDE treatment

(+)-Anti-benzo[a]pyrene-7,8-dihydrodiol-9,10-epoxide (BIU, *Biochemisches Institut für Umweltcarcinogene*, Germany) was dissolved in water-free tetrahydrofuran (THF) supplemented with 5% triethylamine (TEA). Aliquots of the stock solution (10 mM) were snap-frozen in liquid nitrogen and stored at − 80 °C. Treatment of cells with BPDE was performed in cell culture medium without supplements. To keep the THF solvent concentration of the working solutions stable at 0.1%, freshly prepared BPDE stock solutions were diluted (1:1000) in growth medium w/o supplements (termed ‘incomplete growth medium’). If not specified otherwise, cells were treated for 1 h with BPDE at 37 °C, then medium was replaced with pre-warmed complete growth medium and cells were incubated further at the standard conditions until experimental readout.

### Immunochemical detection of BPDE-DNA adducts

#### Immunofluorescence microscopy

HeLa cells were seeded on coverslips in 12-well plates (1 × 10^5^ cells per well). After incubation for 1 day, cells were treated with BPDE in incomplete DMEM for 1 h and washed afterwards thrice for 5 min in PBS. Cells were fixed with methanol:acetone (1:1) for 20 min at − 20 °C, air-dried, and treated with 0.05-M HCl for 5 min on ice. Samples were washed thrice for 5 min in PBS and incubated with RNaseA (100 µg/ml in 150-mM NaCl, 1-mM sodium citrate) for 1 h at 37 °C. Thereafter, cells were incubated consecutively in PBS or EtOH (35%-, 50%-, and 75%) for 3 min each. Next, DNA was denatured in 150-mM NaOH in 70% EtOH for 4 min, and washed twice in PBS. Cells were incubated for 2 min each in 70% EtOH containing 4% formaldehyde, in 50% and 35% EtOH. DNA was stained using Hoechst33342 (200 ng/ml in PBS) for 10 min at room temperature (RT) and subsequently treated with Proteinase K (10 µg/ml in 20 mM Tris, 1-mM EDTA, pH 7.4) for 10 min at 37 °C. Cells were washed again thrice for 5 min in PBS before incubation in 20% FCS (in PBS) for 1 h at RT. Cells were washed thrice in 0.05% Tween 20 (in PBS) for 5 min and incubated with a primary antibody against BPDE-DNA adducts (Santa Cruz, 1:50 in PBS/5% FCS) for 1 h at 37 °C in a humid chamber. Again, cells were washed thrice in 0.05% Tween 20 (in PBS) and then incubated in secondary antibody solution (1:400 GαM-Alexa Fluor 488 in PBS/5% FCS) for 1 h at 37 °C in a humid chamber. Coverslips were washed thrice for 5 min in PBS before they were mounted with Aqua-Polymount. Microscopic images of at least 100 cells were acquired with an Axiovert 200M microscope (Zeiss). Mean fluorescence intensity was determined using the ImageJ software.

#### Slot-blot analysis

One day after seeding of 6 × 10^5^ HeLa cells in 35-mm petri dishes, the medium was removed and exchanged to incomplete DMEM supplemented with BPDE as indicated. Cells were incubated for 1 h at 37 °C, afterwards trypsinized, and pelleted at 200×*g* for 5 min. The pellet was dissolved in 500-µl extraction buffer (100-mM Tris; 200-mM NaCl; 0.2% SDS; 5-mM EDTA, pH 8.0) and 100-µg/ml Proteinase K was added and mixed by inversion. The cell lysate was incubated overnight in a thermomixer (300 rpm; 55 °C) and centrifuged on the next day at 13,000 rpm for 5 min. 500-µl isopropanol were added to the supernatant and incubated for > 1 h at 20 °C. The lysate was centrifuged at 13,000 rpm for 10 min at 4 °C and the resulting pellet was dissolved in 500-µl ice-cold EtOH. The sample was centrifuged again at 13,000 rpm for 5 min at 4 °C, the pellet air-dried, and dissolved in 50-µl TE buffer. Each well of a slot-blot manifold was pre-rinsed with 500-µl MilliQ water and 500-µl 6 × SSC buffer. Prior to sample loading, DNA was denatured by adding NaOH and EDTA to the final concentrations of 0.4 M and 10 mM, respectively. Then, samples were incubated for 10 min at 95 °C, diluted in TE, and applied on the slot-blot manifold. The wells were rinsed again in 500-µl NaOH (0.4 M) and the membrane was washed in 2 × SSC buffer. The air-dried membrane was incubated for 1 h in 5% skim milk in TBS-T. Afterwards, the blot was incubated for 1 h in a BPDE-DNA-specific antibody, diluted 1:400 in blocking buffer, and washed thrice for 5 min in TBS-T before incubation in secondary antibody solution (GαM HRP, 1:2000 in blocking buffer, 1 h). The membrane was washed again in TBS-T and chemiluminescence was detected via ECL reaction.

### PAR detection

HeLa cells were seeded in 60-mm dishes (6 × 10^5^ cells per dish) and cultured for 2 days to obtain a final amount of ~ 6–7 × 10^6^ cells. Medium was replaced with pre-warmed incomplete medium supplemented with or without 10-µM ABT888 30 min prior to BPDE treatment. PAR formation was induced by replacing the medium containing 10- or 50-µM BPDE (0.1 or 0.5% THF, respectively) in pre-warmed incomplete DMEM and incubation took place for 10, 30, 60, or 90 min. After washing with ice-cold PBS, cells were lysed with ice-cold 20% TCA and detached mechanically using a cell scraper. The cell lysate was centrifuged for 4 min at 4 °C at 3000×*g*, the supernatant was discarded, and the pellet washed twice with ice-cold 70% EtOH. Finally, the pellet was dried at 37 °C in a thermo shaker/thermomixer and stored at − 20 °C. PAR detection using isotope dilution mass spectrometry was performed as described previously (Martello et al. 2013; Zubel et al. 2017).

### NAD^+^ cycling assay

NAD^+^ measurements were performed as described previously (Jacobson and Jacobson 1976), with modifications. HeLa cells (1 × 10^5^ cells/well) were seeded in 6-well plates and treated with BPDE in concentrations as indicated in incomplete growth medium for 1 h. BPDE was removed and cells were harvested directly or allowed to recover for 4 or 23 h in complete growth medium. Cell numbers were adjusted to 5 × 10^5^ cells. After centrifugation for 5 min at 4 °C and 200×*g*, the pellets were dissolved in 500-µl ice-cold PBS, and 24-µl cold 3.5-M HClO_4_ were added and mixed thoroughly. The reaction tubes were put on ice for 15 min and centrifuged again for 10 min at 4 °C and 1500×*g*. The supernatant was transferred to new reaction tubes, mixed with 350-µl phosphate buffer (0.33-M K_2_HPO_4_; 0.33-M KH_2_PO_4_; pH 7.5) and incubated on ice for an additional 15 min. Again, the solutions were centrifuged for 10 min at 4 °C and 1500×*g*. The supernatant was recovered and incubated on ice for 20 min. Afterwards, samples were centrifuged (10 min, 4 °C, 1500×*g*) and the supernatant was transferred to fresh reaction tubes. Samples were distributed in technical triplicates on a 96-well plate (40 µl/well) and mixed with 160-µl diluent (0.5 M H_3_PO_4_; 0.5 M NaOH). Immediately before measurement, 5 volumes of Premix solution (0.48 M bicine, pH 8.0; 4-mg/ml BSA; 20-mM EDTA; 2.4-M EtOH; 2-mM MTT) were mixed with one volume phenazine ethosulfate (PES, 40 mM, Santa Cruz) and one volume alcohol dehydrogenase (ADH,1 mg/ml, Sigma Aldrich). The resulting reaction mix (100 µl) was added to every well, mixed thoroughly, and samples were incubated at 30 °C. After 30 min, the absorption was measured using a microplate reader (filterset 550/690 nm, *A*_max_ at 570 nm).

### Alamar blue assay

HeLa cells were seeded in a 96-well plate (100 µl/well; 6000 cells/per well) in technical triplicates or quadruplicates and incubated for 3 h at 37 °C for attachment. Thirty minutes prior to the treatment, medium was changed to fresh growth medium with or without 10-µM ABT888. BPDE was diluted in pre-warmed, incomplete DMEM, and applied to cells. After 1 h at 37 °C, medium was changed again to fresh growth medium. In case of PARP inhibition, ABT888 was present during and after the BPDE treatment. After incubating the cells for 24 h or 45 h, a 10% Alamar blue solution (Thermo Scientific) was added to each well. After additional 4 h, fluorescence was measured using a Varioskan Flash fluorescence reader (Ex.: 535 nm/Em.: 580 nm).

### Annexin V/propidium iodide assay

HeLa cells were seeded in 6-well plates (3 × 10^5^ cells per well) and incubated overnight. PARP inhibition was achieved by treating cells with 10-µM ABT888 in incomplete medium 30 min prior to BPDE treatment. After 1 h, BPDE was removed and the cells were further cultured in growth medium in the presence or absence of ABT888 for 48 h. Afterwards, cells were washed with PBS and harvested by trypsination. Culture medium, PBS used for washing, and the harvested cells were collected and pooled. The cell suspension was centrifuged for 5 min at 1000 rpm, the pellet resuspended in ice-cold PBS, and the total cell number was determined. One million cells were centrifuged for 5 min at 4 °C and 1000×rpm and resuspended in 1-ml Annexin V binding buffer (10-mM HEPES/NaOH, pH 7.4; 140-mM NaCl; and 2.5-mM CaCl_2_). Five microliters of Annexin V-FITC solution were added to 195 µl of cell suspension and incubated in the dark for 15 min. Subsequently, 200-µl PI staining solution (10-mM HEPES/NaOH, pH 7.4; 140-mM NaCl; 2.5-mM CaCl_2_; and 10-µg/ml PI) was added and kept on ice until measurement. Unstained, as well as PI and Annexin V single-stained samples were prepared to establish and calibrate instrument parameters and correct gating. Measurement of 20,000 cells was performed with a BD FACSCalibur and results were analyzed with the FlowJo 8.8.7 software.

### Clonogenic survival assay

HeLa cells were trypsinized and cell numbers were determined in three replicates using a Casy cell counter (Roche). Cells were centrifuged for 5 min at 1000 rpm and resuspended in incomplete medium to obtain a final concentration of 2 × 10^5^ cells/ml. The cell suspension was distributed in 2-ml reaction tubes (1 ml each) and incubated at 37 °C in the presence or absence of 10-µM ABT888. After 10 min, 1 µl of the freshly prepared 1000 × BPDE stock solution was added and mixed carefully by pipetting. Treatment was performed at 37 °C for 30 min. Cell suspensions were further diluted 1:100 in complete growth medium before 1000 cells were seeded in 60-mm petri dishes in the presence or absence of 10-µM ABT888. In an alternative treatment schedule, ABT888-untreated cells were seeded and incubated for 6 h to allow attachment before adding 10-µM ABT888. After incubating the cells for 7 days at 37 °C, they were fixed with 10% PFA for 30 min and stained with 0.1% crystal violet (in PBS) for 45 min. Excessive crystal violet was removed by repeated washing with MilliQ water and the dishes were air-dried and sealed with Parafilm. Colony numbers (> 20 cells/colony) were determined using a binocular (Leica).

### Host cell reactivation assay

To analyze the repair of BPDE-DNA lesions in a cellular, chromatin-independent context, a host cell reactivation assay (HCRA) was performed as described previously (Burger et al. 2010). To this end, human foreskin fibroblasts were left untreated or were treated with 1- or 10-µM ABT888 for 30 min. Thereafter, cells were harvested by trypsination and the resulting cell suspension was divided in two equal aliquots. One aliquot was transfected with a plasmid mix consisting of 3-µg pEGFP and 15-µg pDsRed plasmid (‘preparation 1’). The other aliquot was transfected with a mix of the same plasmid quantities, but the pDsRed plasmid was treated with BPDE (‘preparation 2’). The pDsRed plasmid (500 µg/ml) was treated with 75-µM BPDE in the dark or left untreated. After 3 h of incubation, the plasmids were frozen at − 20 °C and stored until further processing. Transfection was performed in a 4-mm gap cuvette at 0.32 kV and 500 µF with a GenePulser II (Bio-Rad). After electroporation, cells were seeded in 6-well plates in the presence or absence of ABT888. 24 h later, cells were harvested by trypsination and the fluorescence signals were analyzed with a FACSCalibur flow cytometer. Repair capacity was calculated from relative amounts of red fluorescent cells compared to green fluorescent cells between ‘preparation 1’ and ‘preparation 2’ for each treatment. Data were normalized to the untreated control.

### Analysis of reactive oxygen species

HeLa cells were seeded in a 96-well plate (8 × 10^3^ cells/well) and incubated overnight. Medium was exchanged with phenol-red-free DMEM (31053, Gibco). As a positive control, cells were treated with 400 µM of the ROS inducer tert-butyl hydroperoxide (TBHP) for 30 min. As a negative control, cells were additionally treated with 5 mM of the antioxidant N-acetyl cysteine (NAC) for 1 h before TBHP treatment. Samples were treated with 10- or 50-µM BPDE in incomplete phenol-red-free DMEM for periods as indicated in technical triplicates at 37 °C. Thereafter, 4-µM dihydroethidium (DHE) was added, and after 30 min at 37 °C, fluorescence signals (Ex.: 520 nm/Em.: 610 nm) were acquired.

### Cell-cycle analysis

Unsynchronized cell culture: HeLa cells were seeded in 6-well plates (4 × 10^5^ cells/well) and incubated overnight. 30 min prior to BPDE treatment, medium was replaced with fresh growth medium supplemented with or without 10-µM ABT888. BPDE treatment was performed in incomplete medium at 37 °C for 1 h. Thereafter, medium was replaced with complete growth medium and cells were cultured for 24 h. On the next day, cells were harvested, pelleted by centrifugation for 5 min at 1000 rpm, and resuspended in 300-µl cold PBS. The cell suspension was mixed by adding dropwise 700-µl ice-cold ethanol and kept on ice for 20 min or alternatively stored at − 20 °C overnight. Cells were centrifuged for 5 min at 4 °C and 200×*g*, washed with 150-µl ice-cold PBS, and again centrifuged for 5 min at 4 °C and 300×*g*. This was repeated once, before the pellets were resuspended in 30-µl PBS and mixed thoroughly with 120-µl DNA extraction buffer (4-mM citric acid; 0.2-M Na_2_HPO_4_; pH 7.8). After 20 min under gentle agitation, cells were centrifuged for 5 min at 300×*g* and the pellet was resuspended in 200-µl DNA staining solution (20-µg/ml propidium iodide; 0.2-mg/ml DNase-free RNase A in PBS). Incubation was performed for 30 min at RT, before cells were stored on ice in the dark until measurements. The cell-cycle phase was determined by analysis of cellular PI signal as a marker of DNA content. 30,000 cells were measured with a BD FACSCalibur and results were analyzed with the FlowJo 8.8.7 software.

Synchronized cell culture: For cell-cycle synchronization, 3.2 × 10^6^ HeLa cells were seeded in T-75 culture flasks. On the next day, 500-nM nocodazole was added to the normal growth medium and cells were incubated for 12 h to induce a G2/M arrest. To further increase the degree of synchronization, a mitotic shake-off was performed. The supernatant, which contained M-phase cells, was taken off, centrifuged for 5 min at 200×*g*, and washed twice in PBS. Four hundred thousand cells were seeded in 6-well plates (t_0_) and kept at 37 °C. 10 min prior to BPDE treatment, the medium was replaced with DMEM without or supplemented with 10-µM ABT888. 10 h after seeding (t_10_), BPDE treatment was performed in incomplete medium at 37 °C. Afterwards, cells recovered in complete growth medium. At t_11_, t_14_, t_18_, t_24_ t_26_, t_29,_ and t_32_, cells were washed with PBS, harvested, and prepared for cell-cycle analysis as described for unsynchronized cells.

### γH2A.X immunostaining

HeLa cells were seeded in 6-well plates (3 × 10^5^ cells/well) and incubated overnight. 30 min prior to BPDE treatment, cells were treated with 10-µM ABT888 or left untreated. Then, cells were exposed to 50-nM BPDE in incomplete medium in the presence or absence of ABT888. After 1 h, BPDE was replaced with growth medium (supplemented with ABT888 or left untreated) and incubated at 37 °C for periods as indicated. Before harvesting, cells were washed with PBS and 100-µl pre-heated (95 °C) 2 × SDS loading dye was applied to the cells and incubated for 2 min. Cells were detached from the plate with a cell scraper and the cell lysates were collected in 1.5-ml reaction tubes. Immediately thereafter, the solutions were heated again to 95 °C for 5 min. DNA was sheared by repeated passaging through a 27-gauge needle. Cell lysates were heated again for 5 min to 95 °C, and 10 µl of each sample was subjected to a 15% SDS–PAGE and subsequent wet-blotting. The blot was cut in half according to target protein sizes (~ 15 kDa for γH2A.X and ~ 42 kDa for actin) and membranes were incubated in blocking solution (5% skim milk in TBS-T). For actin, γH2A.X-staining membranes were incubated for 1 h in primary antibody solutions [1:50,000 α-actin and 1:2000 α-γH2A.X (MAB1501 and JBW301, Merck) in blocking buffer] and afterwards washed thrice for 5 min in TBS-T. Subsequently, membranes were incubated in secondary antibody solution for 1 h [1:2000 goat-anti-mouse-HRP (DakoCytomation) in blocking buffer], followed by washing thrice with TBS-T for 5 min. Chemiluminescence signals were detected after applying ECL solution (Lumigen) with an ImageQuant LAS 4000 (GE Healthcare).

### Immunofluorescence microscopy

Co-staining of γH2A.X and EdU: HeLa cells were seeded on coverslips in 12-well plates (1 × 10^5^ cells/well) 1 day before the experiment. 20 min before BPDE treatment, cells were incubated in 10-µM EdU, which was also present during BPDE exposure (1 h, 150 nM). Cells were washed thrice with PBS, and subsequently incubated in complete growth medium. At timepoints of 1, 2, 4, and 8 h after damage induction, cells were fixed with 4% PFA/PBS for 20 min at RT. After a 5-min washing step with PBS, samples were treated with 50-mM NH_4_Cl/PBS for 10 min. Cells were washed again with PBS (2 × for 5 min), permeabilized with 0.2% Trition X-100/PBS for 4 min, and washed again (2 × for 5 min in PBS). Blocking occurred in 1% BSA in PBS for 30 min at RT before cells were incubated overnight at 4 °C in primary antibody solution (anti-γH2AX, 1:500 in blocking buffer) in a humid chamber. On the next day, cells were washed consecutively for 5, 10, and 15 min in PBS. Secondary antibody solution [goat-anti-mouse Alexa Fluor 488 (Molecular Probes)] was diluted 1:400 in blocking buffer and cells were incubated for 45 min at RT in a humid chamber. Thereafter, cells were washed again for 5, 10, and 15 min with PBS. To detect the incorporated thymidine analogue EdU, a click reaction was performed with a fluorescent azide (Click-iT Plus Kit, Thermo Scientific) according to the manufacturer’s instructions.

Co-staining of γH2A.X and 53BP1: Sample treatment and preparation was performed as described for the γH2A.X/EdU co-staining with some variations. Here, simultaneous to the first antibody staining with γH2A.X, cells were co-incubated with rabbit anti-53BP1 antibody (1:200, H-300, SataCruz). The same applies for the incubation with the secondary antibody (goat-anti-rabbit Alexa Fluor 568, 1:400, Molecular Probes). Confocal microscopy was performed on a Zeiss LSM780 and > 100 cells per condition were analyzed. Data were evaluated using ImageJ and numbers of γH2A.X foci per cell or their co-localization with foci of 53BP1 were defined using the BIC Macro Toolkit (BIC, University of Konstanz).

### HPRT mutagenicity assay

Pre-existing HPRT mutant cleansing (HAT selection): Three million CHO cells were seeded in T-160 cell culture flasks. The next day, medium was replaced with complete growth medium supplemented with HAT (hypoxanthine–aminopterin–thymidine) for *HPRT* mutant removal. Cells were cultured in HAT selection medium for 72 h. Thereafter, HAT medium was replaced with HT medium (hypoxanthine–aminopterin) and cells were allowed to recover for 48 h.

BPDE treatment and phenotypic expression: Cells were re-seeded in 6-well plates (3 × 10^5^ cells/well) and allowed to adhere overnight. 30 min before BPDE treatment, cells were treated with 10-µM ABT888 or left untreated. CHO cells were incubated at 37 °C in incomplete medium supplemented with BPDE in the presence or absence of ABT888. After 1 h, BPDE was removed, and cells were washed once with PBS and incubated for 23 h in fresh growth medium. On the next day, cell numbers were readjusted to 3 × 10^5^ cells/well and cells were incubated for 11 days (w/ or w/o ABT888), with sub-culturing every other day. Afterwards, cells were harvested and cell numbers were determined. A number of 2 × 10^5^ cells were re-seeded in selection medium (supplemented with 40-µM 6-thioguanine, 6-TG). Mutant selection went on for a period of 8 consecutive days, during which medium and 6-TG were refreshed once after 4 days of culturing. Simultaneously, plating efficiency (PE = mean colony number/ seeded cells) was analyzed by seeding defined numbers of CHO cells in technical triplicates and culturing without selection pressure. If applied, 10 µM of ABT888 was present always both in mutant selection and plating-efficiency media. After 8 days, cells were carefully washed once with PBS and fixed with 10% PFA for 30 min. Colonies were stained with 0.05% crystal violet for 30 min and repeatedly washed with MilliQ water. The plates were air-dried and sealed with parafilm and colonies were counted. Cell clusters with more than 20 cells in diameter were defined as countable colonies. Mutant frequency (MF) was calculated as mean colony number (selective conditions)/(PE × 2 × 10^5^).

## Results

The previous studies analyzed the role of PARP1 in NER in response to UV-light-induced DNA damage (Pines et al. 2012; Robu et al. 2013, 2017; Vodenicharov et al. 2005). However, whether and how PARP1 and PARylation are involved in the cellular response to bulky DNA lesions caused by chemical compounds such as B[a]P metabolites is largely unexplored. The prime purpose of this study was to investigate the role of PARP1 and PARylation in BPDE-induced genotoxic stress response and to address the underlying cellular mechanisms.

### BPDE induces a cellular PARylation response

**Fig. 1:**
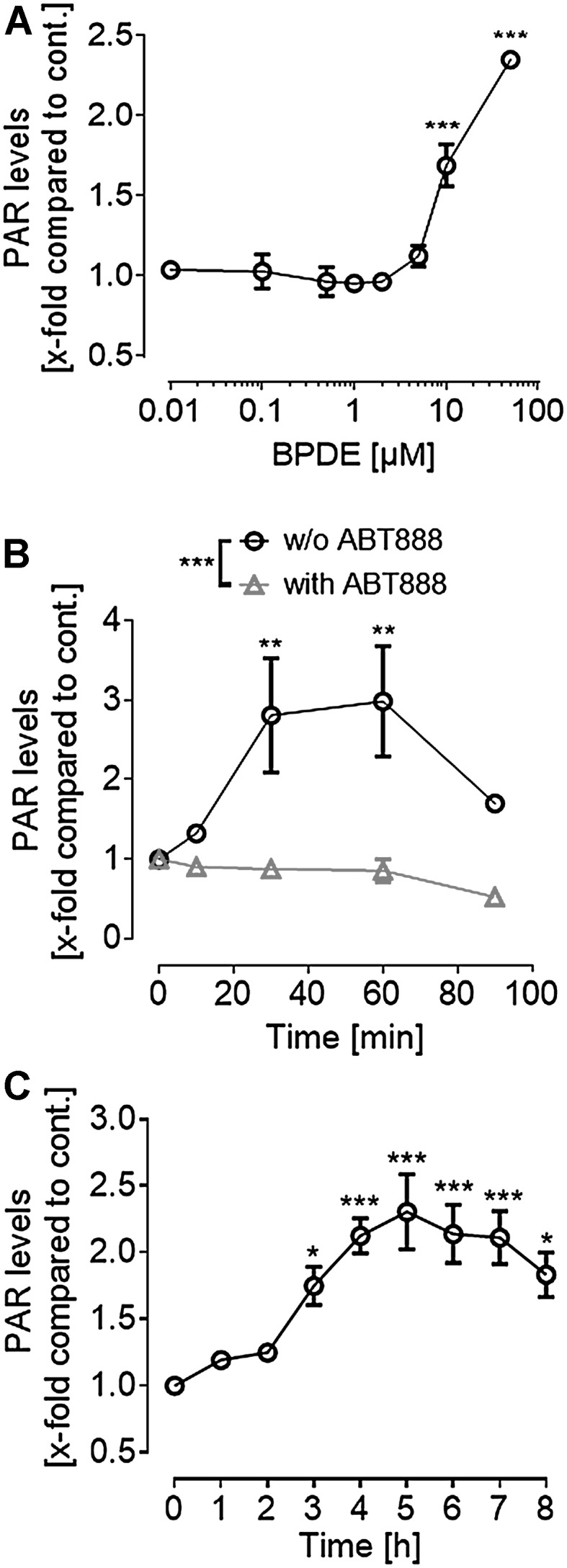
LC–MS/MS analysis of BPDE-induced PAR formation in HeLa cells. **a** Dose–response analysis of BPDE-induced PAR formation. Cells were treated for 1 h with BPDE in concentrations as indicated. Afterwards, PAR levels were determined via LC-MS/MS. **b** Short-term time series of PAR formation after treatment with 50 µM of BPDE. Cells pre-incubated in 10-µM ABT888 showed no PARylation in response to BPDE treatment. **c** Long-term time series of PAR formation after 10-µM BPDE treatment. PAR levels continuously increased for the first 5 h after damage induction. Data represent means ± SEM (*n* = 3) normalized to untreated and solvent control, respectively. Statistical evaluation was performed using one-way **a, c** or two-way ANOVA, **b** analyses followed by Sidak’s multiple comparison testing. *p < 0.05, **p < 0.01, ***p < 0.001. For exemplary LC–MS/MS chromatograms, refer to Suppl. Figure 2

After verifying that BPDE indeed causes DNA adducts in treatment conditions as applied in this study (Suppl. Figure 1), we tested if BPDE can induce PARylation in HeLa cells using a highly sensitive bioanalytical method based on isotope dilution mass spectrometry (LC-MS/MS) (Martello et al. 2013; Zubel et al. 2017) (Fig. 1). Since we recently generated HeLa cells with genetic *PARP1* deletion (mutation) via TALEN-mediated gene targeting (Rank et al. 2016), this cell line was chosen as a model system. In a dose–response experiment, cells were treated with BPDE for 1 h and PAR levels were determined via LC-MS/MS. After exposure to ≥ 10-µM BPDE, a significant increase in PAR formation was observed (Fig. 1a). When treating cells with 50-µM BPDE in a short-term time series, PAR levels started to rise after 10 min, reached a significant threefold induction after 30 min, and declined afterwards by 90 min. ABT888 was used as a specificity control and, as expected, completely inhibited BPDE-induced PAR formation (Fig. 1b). Exemplary LC-MS/MS chromatograms are shown in Suppl. Figure 2. When performing a long-term time series after treatment of cells with 10-µM BPDE, a steady increase of PAR levels was observed reaching a plateau phase after 4–5 h (Fig. 1c). Thereafter, the PAR signal decreased slowly until 8-h post treatment. These LC-MS/MS analyses demonstrate—with full chemical specificity—that BPDE induces a cellular PARylation response.

### BPDE exposure affects cellular NAD^+^ levels

**Fig. 2:**
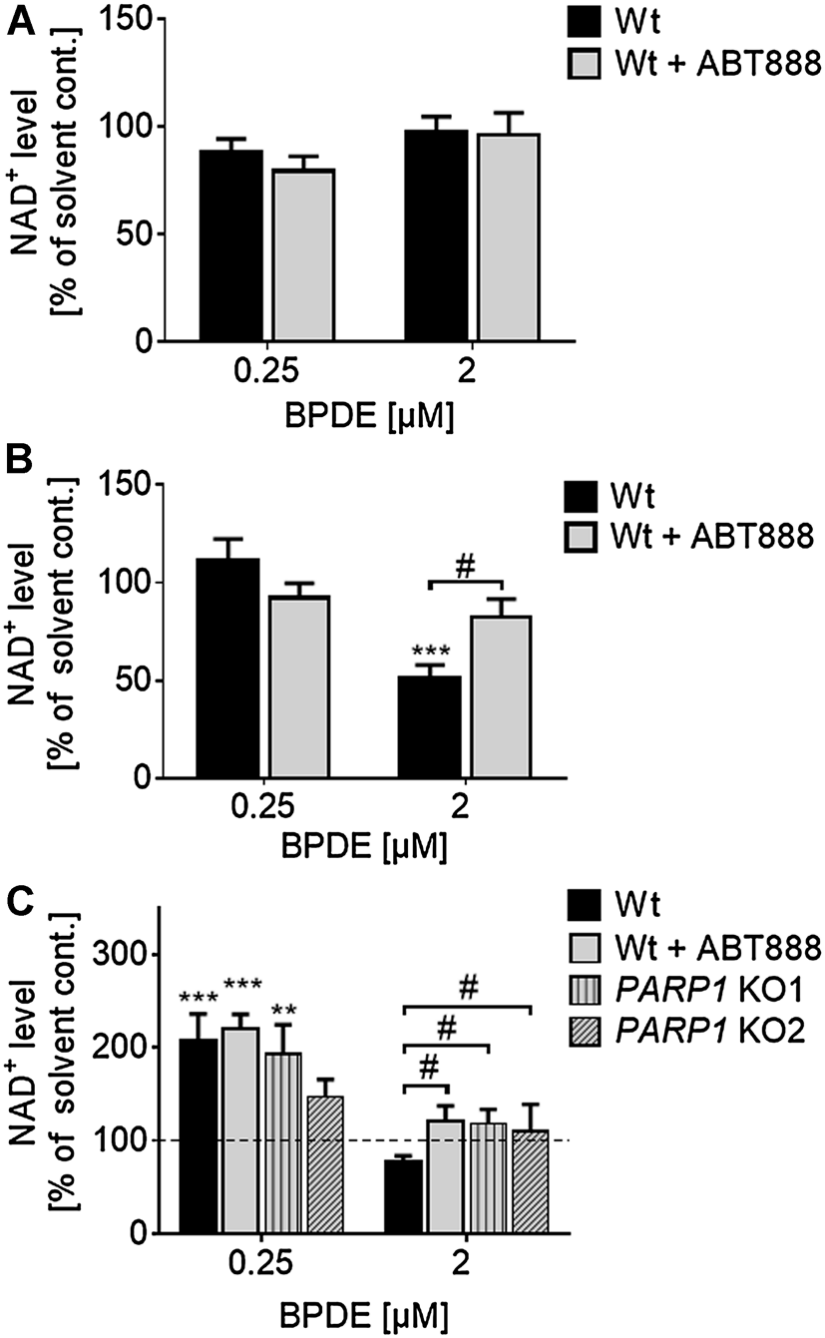
Analysis of cellular NAD^+^ levels upon BPDE treatment. NAD^+^ levels of HeLa cells were determined by an enzymatic NAD^+^ cycling assay (Jacobson and Jacobson 1976). **a, b** Cells were treated with BPDE for 1 h in concentrations as indicated and NAD^+^ levels were analyzed either directly (**a**) or 4 h (**b**) after treatment. 4 h after treatment with 2-µM BPDE, NAD^+^ levels decreased significantly, which could be inhibited by PARP inhibition (**b**). **c** Cells were treated with BPDE for 1 h. 23 h later, NAD^+^ levels were analyzed. After treatment with 0.25-µM BPDE, NAD^+^ levels increased significantly independent of PARP inhibition or genetic deletion of *PARP1*. In contrast, after treatment with 2-µM BPDE, NAD^+^ levels dropped in HeLa Wt cells, but remained unchanged in HeLa *PARP1* knockout (KO) cells. Data were normalized to solvent control and represent means ± SEM of ≥ 3 independent experiments; each performed in technical triplicates. Statistical evaluation was performed using two-way ANOVA analysis followed by a Sidak’s multiple comparison test (*) or using non-parametric two-tailed *t* tests (#). ^#^ *p* < 0.05, **p < 0.01, ***p < 0.001

PAR formation relies on its substrate NAD^+^, which can lead to a significant depletion of NAD^+^ pools in case of strong or long-lasting PARP stimulation. In severe cases, this can result in a metabolic catastrophe and energy crisis (Fouquerel and Sobol 2014). To analyze if BPDE-induced PARylation affects cellular NAD^+^ levels, a quantitative NAD^+^ cycling assay was performed (Fig. 2). Unlike the PARylation response induced by H_2_O_2_ (Rank et al. 2016), PARP activation upon BPDE treatment occurred with slower kinetics, but was lasting for several hours (Fig. 1c). Thus, cells were treated with BPDE for 1 h and NAD^+^ levels were determined either directly or 4 and 23 h after BPDE treatment. Neither concentration of BPDE changed NAD^+^ levels directly after treatment (Fig. 2a). Yet, when cells were treated with 2-µM BPDE, NAD^+^ levels declined by 50% within 4 h after exposure. The decrease of NAD^+^ could be rescued almost completely when cells were incubated with ABT888. Treating cells with a lower concentration of 250-nM BPDE had no influence on cellular NAD^+^ levels even 4 h after treatment (Fig. 2b). Interestingly, 23 h after treatment with 2-µM BPDE, NAD^+^ levels were still reduced by 20% (Fig. 2c), suggesting a long-lasting moderate stimulation of PARP1 activity. Consistent with the notion that decreases in NAD^+^ levels result from PARP1 activation, NAD^+^ levels were rescued by PARP inhibitor treatment as well as genetic deletion of *PARP1*. Strikingly, when cells were exposed to a lower concentration of 250-nM BPDE, a twofold increase of NAD^+^ levels as compared to solvent control was observed 23 h after treatment, irrespective of PARP inhibitor treatment or genetic ablation of *PARP1* (Fig. 2c). These results demonstrate that BPDE exposure significantly influences cellular NAD^+^ metabolism in a complex and at least in part in a PARP1-dependent manner.

### PARP inhibition protects HeLa cells from short-term BPDE-induced toxicity

**Fig. 3:**
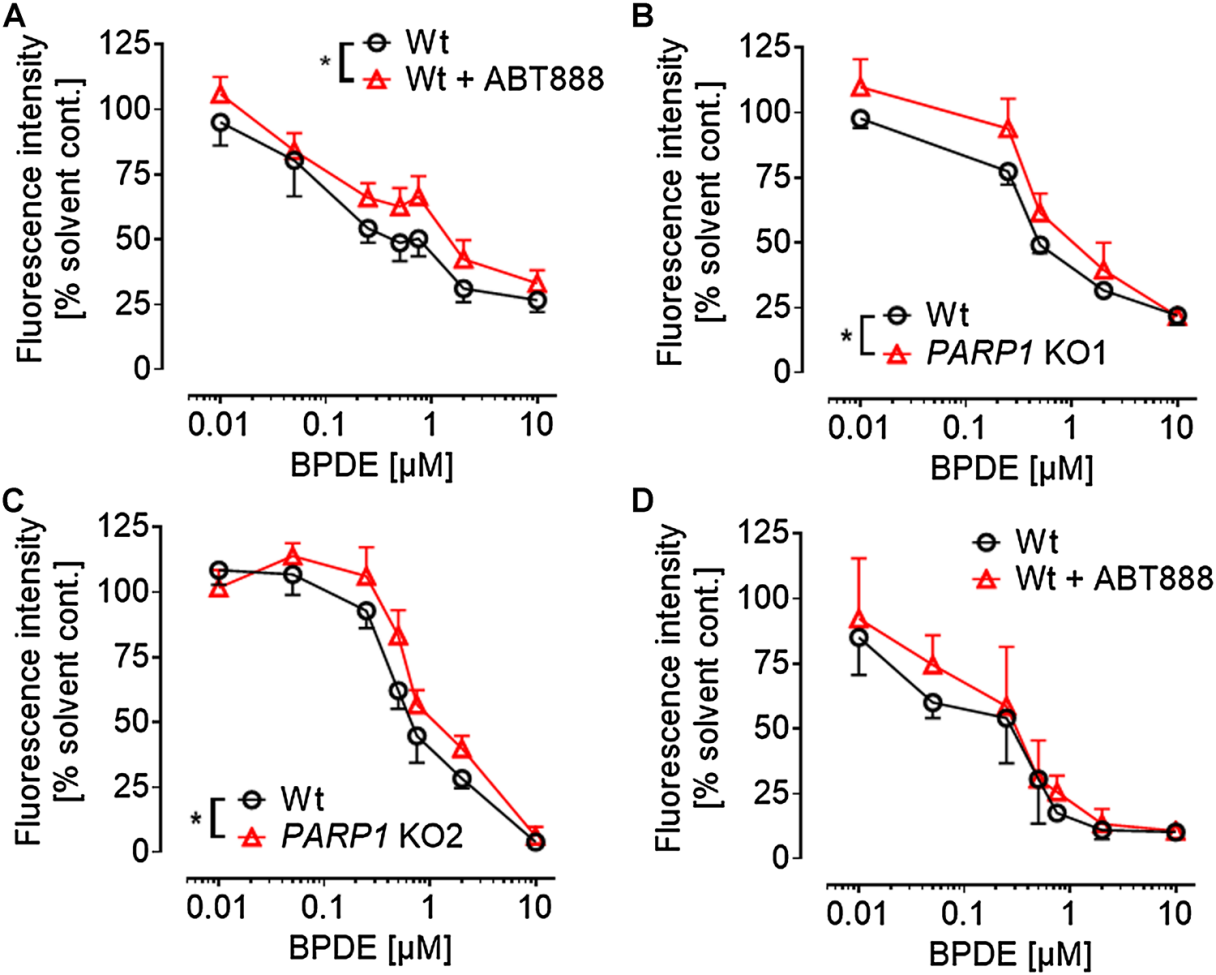
Genetic *PARP1* ablation or PARP inhibition protects from BPDE-induced short-term cytotoxicity. Alamar Blue assays were performed with HeLa cells to analyze the influence of PARP1 activity on cell proliferation and metabolic activity 24 h or 45 h after BPDE treatment. **a** Cells were treated with increasing concentrations of BPDE and incubated for 24 h before analyzing fluorescence intensities. ABT888 (10 µM) partially protected from BPDE-induced cytotoxicity. **b, c** Genetic ablation of *PARP1* also protected cells from BPDE-induced cytotoxicity. Alamar Blue assays were performed 24 h after BPDE treatment. Both *PARP1* knock-out cell lines (KO1 and KO2) were significantly more resistant to BPDE-induced toxicity compared to HeLa Wt cells. **d** When the same assay was conducted 45 h after BPDE treatment, the protective effect of ABT888 was diminished and did not reach statistical significance. For solvent control, see Suppl. Figure 3. Data represent means ± SEM of ≥ 3 independent experiments, and each performed in ≥ 3 technical replicates, normalized to solvent control. Statistical evaluation was performed using two-way ANOVA analysis followed by Sidak’s multiple comparison testing. *P < 0.05

To test if PARP activation and the alterations in NAD^+^ levels influence BPDE-induced toxicity, we tested if co-treatment of BPDE and ABT888 affects cell proliferation and/or cytotoxicity. As expected, when using the Alamar Blue assay to analyze cell proliferation and metabolic activity, we observed a significant decline in cellular activity 24 h after BPDE treatment at concentrations ≥ 100 nM. Interestingly, this decline in cellular activity was slightly, yet statistically significant, inhibited by ABT888 treatment (Fig. 3a). Consistent with these results, the same trend was observed in two independently generated HeLa cell lines carrying a genetic deletion of *PARP1* (Fig. 3b, c). Notably, the protecting/inhibitory effect of ABT888 was only visible 24 h after BPDE treatment, since 45 h upon BPDE exposure, no significant difference of BPDE-induced cytotoxicity was observed between ABT888-treated and non-treated cells (Fig. 3d). ABT888 treatment or genetic deletion of *PARP1* alone had no effect on cellular activity in the Alamar Blue assay (Suppl. Figure 3). Since PARP activation and NAD^+^ depletion may result in enhanced cell death (Fouquerel and Sobol 2014), we next analyzed potential effects of BPDE and ABT888 co-treatment on cellular apoptosis and necrosis by Annexin V/PI staining (Suppl. Figure 4). Significant BPDE-induced cell death was observed at concentrations ≥ 2 µM at 45 h after treatment. In contrast to results obtained with the Alamar Blue assay, no effect of ABT888 co-treatment was observed. These results suggest that PARP activation and changes in NAD^+^ levels were sufficient to affect the influence of BPDE on cell proliferation; however, changes in NAD^+^ levels were too mild to significantly influence BPDE-induced cell death.

### Ablation of PARylation sensitizes cells to BPDE treatment in the clonogenic survival assay

**Fig. 4:**
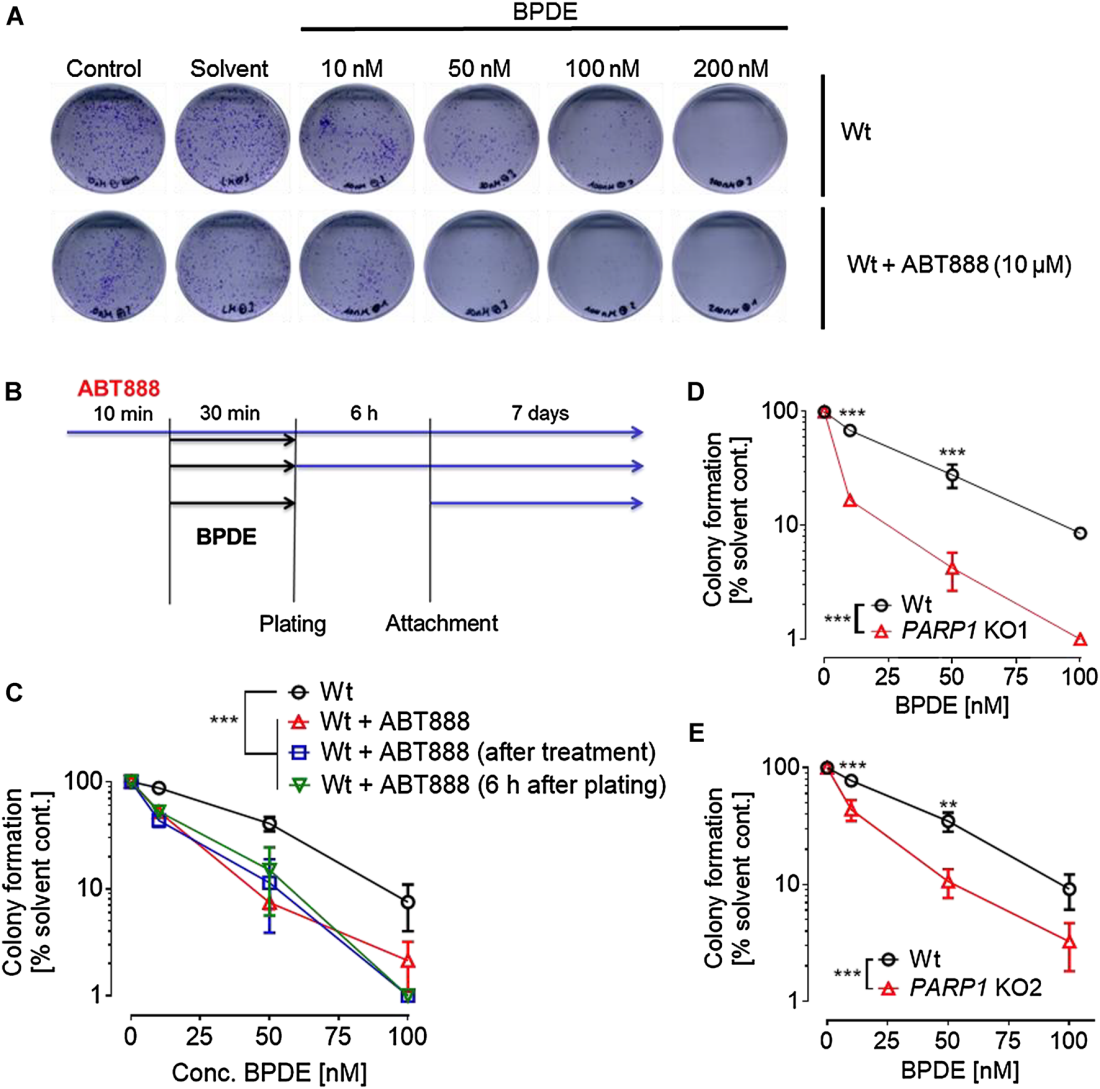
PARP inhibition or genetic *PARP1* ablation potentiates BPDE’s long-term toxicity. HeLa Wt or *PARP1* knockout (KO1/KO2) cells were treated with increasing concentrations of BPDE for 30 min and clonogenic survival assays were performed by incubating cells for 7-day posttreatment. **a** Representative cell culture dishes showing results from a clonogenic survival assay performed with cells treated with BPDE in concentrations as indicated. In all experiments, no colony formation was observed upon 200-µM BPDE treatment. **b** Scheme showing the different treatment schedules to test for the importance of timing of PARP inhibition and BPDE exposure. Three different treatment schedules were tested. **c** Quantification of clonogenic survival assays described in (**b**). Red: ABT888 was present before, during, and after BPDE treatment. Blue: ABT888 was added directly after the treatment. Green: ABT888 was added 6 h after BPDE exposure. No major influence on BPDE’s toxicity could be observed when altering the timing of PARP inhibition. All three ABT888 approaches showed significantly less colonies than the ABT888-untreated one while displaying only minor differences in-between the different ABT888 treatment schedules. **d, e** Similar to pharmacological PARP inhibition, genetic *PARP1* ablation also potentiated BPDE’s induced long-term cytotoxicity. Data represent means ± SEM of ≥ 3 independent experiments and each performed in technical triplicates, normalized to solvent control. For control assays, see Suppl. Figure 5. Statistical evaluation was performed using two-way ANOVA analysis followed by Sidak’s multiple comparison testing. *p < 0.05, **p < 0.01, ***p < 0.001. (Color figure online)

Colony formation assays were performed to test for functional implications of PARylation in BPDE-induced long-term toxicity. As expected, BPDE treatment alone had a strong influence on colony formation. A concentration of 100-nM BPDE reduced the number of colonies by 80–90%, while a concentration of 200-nM already completely inhibited colony growth (Suppl. Figure 5 and Fig. 4). The solvent THF itself had no influence on colony formation of HeLa cells. Since ABT888 treatment alone led to smaller and fewer colonies, independent of BPDE treatment (Suppl. Figure 5), colony numbers were normalized to corresponding solvent controls. ABT888 was applied 10 min before, directly after, or 6 h after plating of BPDE-treated cells. ABT888 treatment directly after exposure was performed with the purpose to exclude any potential unknown chemical interactions between ABT888 and BPDE. ABT888 treatment 6 h after plating was conducted to analyze potential effects of co-treatment with BPDE and ABT888 on cell attachment. PARP inhibition strongly sensitized cells to BPDE and fewer colonies were formed at any BPDE concentration tested, irrespective of the timepoint when ABT888 was applied. The results observed upon treatment with ABT888 were verified using HeLa *PARP1* KO cell lines. Both *PARP1* KO cell lines showed comparable responses as seen upon ABT888 treatment, thus genetic deletion of *PARP1* also strongly potentiated BPDE’s cytotoxic effects (Fig. 4d, e).

### PARP inhibition does not influence the repair of BPDE-DNA lesions in an HCRA

Since colony formation assays (Fig. 4) revealed an important role for PARP activity in the cellular response to BPDE exposure, we tested if PARP activity may play a direct role in the removal of BPDE-DNA adducts using a previously developed host cell reactivation assay (HCRA) (Burger et al. 2010) (Suppl. Figure 6). While 24 h after transfection, the cellular DNA repair machinery restored about 60% of the transfected cells carrying the BPDE-treated plasmids, no effect of PARP inhibitor treatment was evident. These results suggest that PARylation may play no or only a minor role in the direct removal of BPDE-induced DNA adducts in a chromatin-independent repair environment.

The previous studies reported that reactive oxygen species (ROS) can be formed upon B[a]P treatment during CYP450-dependent metabolism (Briede et al. 2004). To exclude that BPDE itself may trigger ROS formation in HeLa cells, which could lead to an indirect activation of PARP1, we performed a DHE-based assay to analyze potential cellular ROS formation. As it is evident from Suppl. Figure 7, no or only minor changes in ROS levels were observed upon treatment of cells with BPDE in concentrations of up to 50 µM, which is consistent with results from a recent report (Christmann et al. 2016).

### PARylation deficiency mildly affects cell-cycle progression in response to BPDE exposure

To test if the sensitization effect of ABT888 in the colony survival assay may have been caused alterations in cell-cycle progression, we employed PI staining coupled to flow cytometric analysis.

First, unsynchronized cells were treated with or without ABT888 in combination with increasing doses of BPDE. After 24 h, a cell-cycle analysis was performed (Suppl. Figure 8). BPDE caused a G2 arrest (right panel) in a dose-dependent manner. The co-incubation with ABT888 enhanced this effect when cells were treated with 100-nM BPDE. The number of cells in S phase did not appear to be affected, neither by BPDE treatment alone nor by BPDE-ABT888 co-treatment. When cells were exposed to higher doses of BPDE, no significant differences between PARP-inhibited and non-inhibited cells could be observed. Doses higher than 0.5-µM BPDE caused a very strong intra-S-phase arrest independent of PARP inhibition (data not shown).

**Fig. 5:**
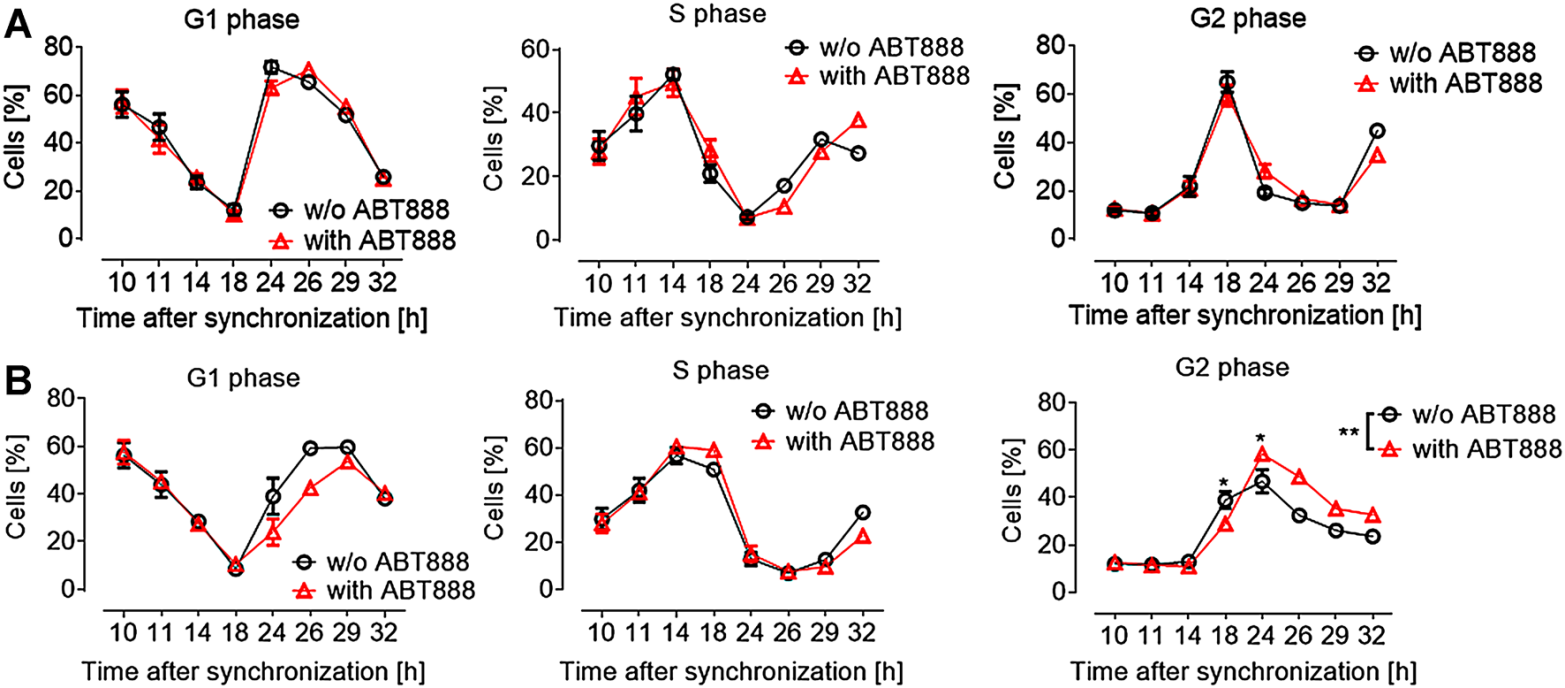
PARP inhibition affects BPDE-induced cell-cycle delay. **a** PARP inhibition in the absence of BPDE had no significant influence on cell-cycle progression within the period analyzed. **b** BPDE treatment (at *t* = 10 h, 0.1 µM for 1 h) of synchronized HeLa cells caused a G2-phase arrest. Additional PARP inhibition further enhanced G2-phase arrest, resulting in decreased cell numbers re-entering the cell cycle. **a, b**. Data represent means ± SEM of ≥ 3 independent experiments (*n* = 1 for *t*_26–32_), normalized to solvent control. Statistical evaluation was performed using two-way ANOVA analysis followed by Sidak’s multiple comparison test. *p < 0.05, **p < 0.01. Results from unsynchronized cells are shown in Suppl. Figure 8

To clarify potential PARP-dependent effects on cell-cycle progression, cells were synchronized before BPDE exposure (Fig. 5). At the timepoint of BPDE treatment, cells were at the border between G1 (60%) and S phases (30%). Figure 5a shows the progression of cells through the cell cycle without BPDE treatment (solvent control). In this case, PARP inhibition alone had no influence on cell-cycle progression. However, as it is evident from Fig. 5b, BPDE treatment led to a cell-cycle arrest in G2 phase, starting 8 h after exposure (Fig. 5b). ABT888 treatment had a statistically significant effect on cell-cycle progression by enhancing the proportion of cells in G2 phase 14 h after BPDE treatment.

### BPDE treatment of PARP1-deficient cells leads to an accumulation of DSB

#### PARylation-deficient cells display increased γH2AX levels

**Fig. 6:**
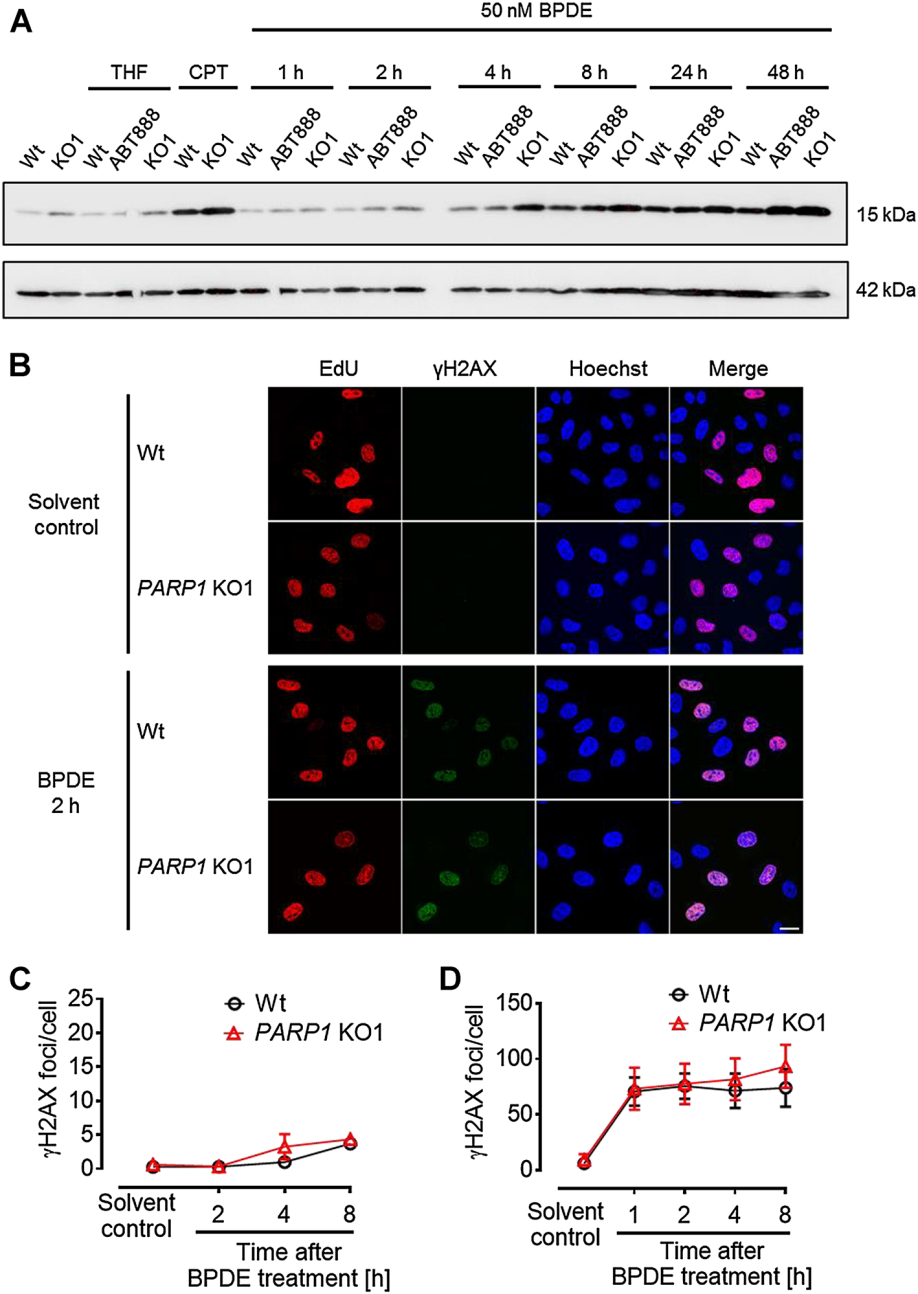
S-phase cells immediately responded to BPDE with increased γH2A.X signaling. **a** HeLa Wt and *PARP1* KO1 cells were treated with 50-nM BPDE for 1 h. After periods as indicated, cells were lysed and subjected to SDS–PAGE and subsequent immunoblotting to detect γH2A.X. Already early after BPDE-dependent damage induction, an increase in γH2A.X signaling could be observed. Signal intensity steadily increased until 2 days after BPDE treatment. The absence of PARP activity (10-µM ABT888) or genetic deletion of *PARP1* further enhanced this damage response. Shown is one representative experiment out of three independent experiments. Actin served as loading control. **b–d** 20 min before as well as during BPDE treatment (150 nM), cells were pulse-labeled with EdU. S-phase cells were identified by means of EdU incorporation. **b** Representative images of immunofluorescence-based detection of γH2A.X signaling in EdU-positive and EdU-negative cells. The scale bar represents 20 µm. **c** Quantification γH2A.X foci in EdU-negative cells. Only a minor increase of γH2A.X foci numbers could be detected 4–8 h after BPDE exposure. **d** In S-phase cells, foci numbers immediately increased upon BPDE treatment. Note the different scale of the *y*-axis. **c, d** Data represent means ± SEM of three independent experiments

The sensitization of cells to BPDE exposure by ABT888 treatment in the clonogenic survival analysis suggested a role of PARP1 within the BPDE-induced replication stress response. Phosphorylation of the core histone H2A.X on serine 139 (γH2A.X) is a sensitive indicator for both DNA damage and replication stress (Szilard et al. 2010). The γH2A.X signal was followed in a time-course experiment after BPDE treatment (Fig. 6a). In untreated as well as in solvent-treated cells, only a weak γH2A.X signal could be detected, indicating no influence of the solvent itself on γH2A.X levels. However, in both controls (untreated and solvent control), the absence of PARP activity resulted in a slight increase in γH2A.X signals even without DNA damage induction. CPT treatment, which served as positive control, resulted in strongly elevated levels of γH2A.X compared to the untreated controls, again displaying stronger signal intensities for *PARP1* KO cells. Exposure of cells to BPDE resulted in continuously increasing amounts of γH2A.X. Already, 2–4 h after treatment, an increase in signal intensity could be observed, which further rose until 48 h after BPDE exposure. The absence of PARP activity visibly amplified the signal intensity for γH2A.X further, with *PARP1* KO1 cells showing even stronger signals than the ABT888-treated samples (Fig. 6a).

To discriminate/determine whether the γH2A.X signaling was a direct response to the BPDE-DNA damage or an indirect result due to BPDE-induced replicative stress, an immunofluorescence analysis of replicating as well as non-replicating cells was performed. Cells were pulse-labeled with EdU, which is incorporated into the DNA during S phase, and were simultaneously treated with BPDE. Thereafter, both substances were removed and cells were cultured further. This setup allowed to discriminate between cells which were in S phase (EdU positive) and all other cell-cycle phase cells (EdU negative) at the time of BPDE treatment (Fig. 6b). Staining for γH2A.X clearly revealed that γH2A.X signaling was due to BPDE-induced replication stress. Cells which were in S phase at the time of BPDE exposure immediately responded strongly to BPDE treatment, giving rise to an average of 70–80 foci per cell. In contrast, during the first 8 h after BPDE exposure, only a moderate increase of γH2A.X foci could be observed in EdU-negative cells, probably marking cells which have not entered replication (Fig. 6c, d). The finding that the γH2A.X response strongly lagged the BPDE exposure, suggested that the underlying cause is not the BPDE-DNA lesions as such, but their faulty procession during replication. If BPDE itself triggered the DDR, a faster response would be expected. Interestingly, the loss of PARP1 protein did not significantly alter the number of γH2A.X foci in immunofluorescence analysis (Fig. 6c, d).

**Fig. 7:**
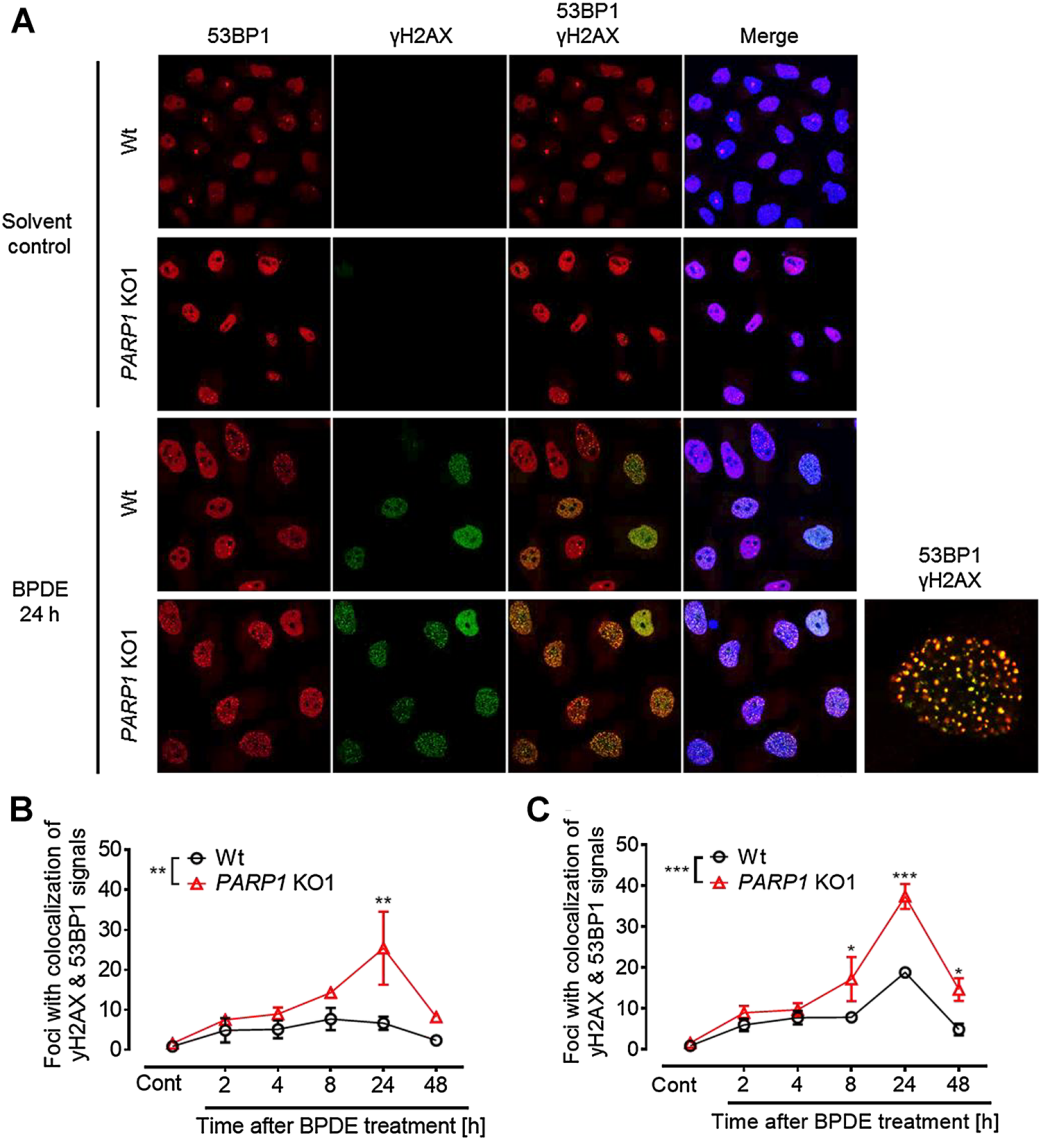
PARP1 deficiency sensitizes cells to BPDE-induced DSB formation. HeLa Wt and *PARP1* knockout (KO1) cells were exposed to BPDE, and at the timepoints indicated, immunofluorescence-based detection of 53BP1 (red channel) and γH2A.X (green channel) was performed. **a** Representative images of cells exposed to 150-nM BPDE. On the bottom right, a digitally magnified *PARP1* KO1 cell is displayed, demonstrating co-localization of 53BP1 and γH2A.X foci. **b** Quantification of co-localization of γH2A.X and 53BP1 foci in cells treated with 50-nM BPDE. **c** Quantification of co-localization of γH2A.X and 53BP1 foci in cells treated with 150-nM BPDE. **b, c** *PARP1* knockout strongly enhanced the numbers of 53BP1 and γH2AX foci co-localization. Data represent means ± SEM of three independent experiments. Statistical evaluation was performed using two-way ANOVA analysis followed by Sidak’s multiple comparison testing. *p < 0.05, **p < 0.01, ***p < 0.001. (Color figure online)

Probably, the most serious outcome of replication stress is the collapse of stalled replication forks and the formation of DSBs. Having observed increased levels of γH2A.X in PARylation-deficient cells compared to Wt cells after BPDE exposure (8–24 h) in Western blot analyses (Fig. 6a), a double staining of γH2A.X and 53BP1 was performed to analyze the impact of PARylation on replication stress-induced DNA double-strand breaks (DSBs) (Fig. 7). 24 h after treatment with 150-nM BPDE, a pronounced co-localization of γH2A.X and 53BP1 was observed, which is indicative of DNA double-strand break formation. When these experiments were performed with a *PARP1* KO cell line, even after low-dose treatment with 50-nM BPDE, a significant increase in γH2A.X/53BP1 was evident (Fig. 7b), which further increased to an average of ~ 37 foci per cell when cells were treated with 150-nM BPDE (as compared to 19 γH2A.X/53BP1 foci in HeLa Wt cells) (Fig. 7c). These results demonstrate that BPDE treatment in the absence of PARP activity led to DNA double-strand breaks specifically in S-phase cells, which are indicative of collapsed replication forks.

### Inhibition of PARylation increases the mutagenicity of BPDE

**Fig. 8:**
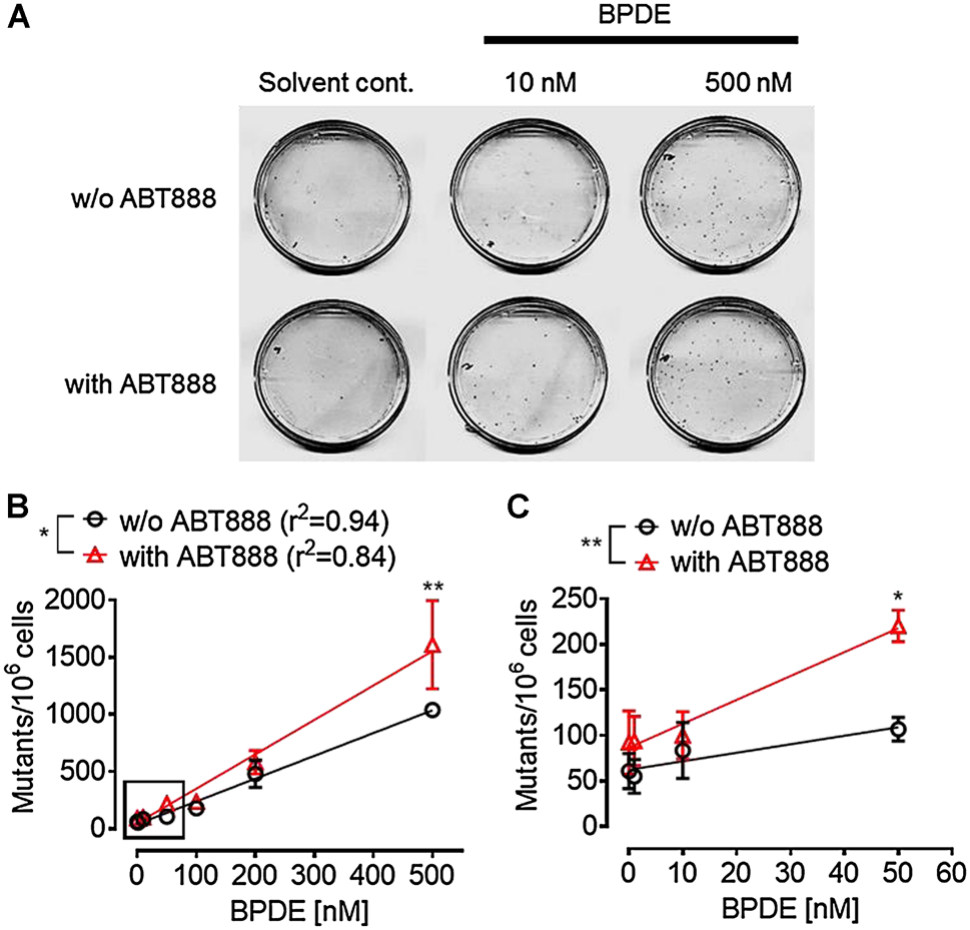
PARP inhibition potentiates BPDE-induced mutagenicity. An *HPRT* mutagenicity assay with BPDE-treated CHO cells was performed in the absence or presence of 10-µM ABT888. **a** Representative cell culture dishes of the *HPRT* assay. A BPDE dose-dependent increase in colony numbers (mutant frequency) was observed. PARP inhibition further increased BPDE-induced mutagenicity. **b, c** Quantification of **(a**) increasing concentrations of BPDE resulted in increased numbers of mutations of the *HPRT* gene. **b** BPDE treatment with concentrations of up to 500 nM. **c** Magnification of insert in B showing data of the low-dose range, with BPDE concentrations of up to 50 nM. When PARP activity was inhibited, an even higher mutation rate was observed. Data represent means ± SEM of four independent experiments. Statistical evaluation was performed using two-way ANOVA analysis followed by Sidak’s multiple comparison testing. *p < 0.05, **p < 0.01

BPDE is a highly mutagenic substance (Newbold and Brookes 1976). To address the question whether PARP activity affects BPDE’s mutagenic potential, an *HPRT* mutation assay was performed (Fig. 8). Consistent with results from the colony formation assays in HeLa cells, ABT888 treatment of CHO cells also resulted in smaller colonies. As expected, increased BPDE concentrations strongly enhanced the mutation frequency in CHO *HPRT* genes (Fig. 8) leading to ~ 1000 surviving mutants per 1 million cells at a concentration of 500-nM BPDE. PARP inhibition itself, in the absence of BPDE treatment, had little influence on the mutation load observed in the *HPRT* assay. In contrast, when cells were treated with ABT888 in addition to BPDE, significantly enhanced numbers of mutant colonies were observed, i.e., ~ 1,500 mutant colonies per 1 million cells, indicating an increased mutagenic potential of BPDE in the absence of PARylation. Since PARylation appeared to be of little importance for direct BPDE-DNA lesion repair (Suppl. Figure 6), this increase is likely to be a direct response to potential replicative stress in PARylation-deficient cells.

## Discussion

Benzo[a]pyrene is a potent and widespread environmental carcinogen and serves as lead model substance to study the response of cells to chemical-induced, bulky DNA adducts that are repaired by NER (Angerer et al. 1997, EPA 2006; Kim et al. 2013; Madureira et al. 2014; Piberger et al. 2017). While there is ample evidence that PARP1 participates in the removal of UV-induced DNA damage during NER (Fischer et al. 2014; Pines et al. 2012; Purohit et al. 2016; Robu et al. 2013, 2017; Vodenicharov et al. 2005), the understanding of how PARP1 participates in genotoxic stress response to chemical-induced, bulky DNA adducts is understood incompletely. Two previous studies provided initial insight into the role of PARylation in B[a]P-induced genotoxic stress. (Lin and Yang 2008; Tao et al. 2009). On one hand, Lin and Yang reported that treatment of HepG2 cells with micromolar concentrations of B[a]P led to NAD^+^ consumption and PARP-dependent cell death (Lin and Yang 2008). On the other hand, Tao et al. reported that B[a]P treatment in micromolar concentrations caused more DNA strand breaks in human bronchial epithelial cells depleted for PARP1 as compared to Wt cells, whereas no PARP1-dependent effect on BPDE-induced cytotoxicity was observed. Moreover, PARP1-depleted cells showed a delay in strand break repair (Tao et al. 2009). While these studies provided first evidence that PARylation plays a role in B[a]P-induced genotoxic stress, the detailed mechanisms of how PARylation is involved herein remained unclear. Since the use of B[a]P as a test compound can lead to ROS formation during xenobiotic metabolism (Briede et al. 2004), it cannot be excluded that in these studies, PARP is activated indirectly by ROS-induced DNA damage. In the present study, we took up the question on the role of PARylation in B[a]P genotoxicity by applying its active metabolite BPDE. In agreement with a recent study (Christmann et al. 2016), we observed no or only minor ROS formation when using BPDE as a genotoxic agent, thus rendering it possible to study PARylation-dependent effects specifically for BPDE-DNA adducts. With this, we now provide a comprehensive molecular toxicological analysis on the role of PARP1 and PARylation in BPDE-induced genotoxic stress by employing genetic and pharmacological approaches in combination with a broad spectrum of toxicological and molecular endpoints. As an experimental model system, we used HeLa wild-type cells treated with PARP inhibitor or untreated as well as HeLa *PARP1* knock-out cells, which have been recently generated using TALEN-mediated gene targeting (Rank et al. 2016).

We demonstrate here for the first time that BPDE directly induces a sustained PARylation response, using a highly sensitive LC-MS/MS approach (Fig. 1). The overall dynamics in PAR formation upon BPDE treatment differ considerably from other genotoxins, such as H_2_O_2_. While H_2_O_2_ causes a strong, but fast PARylation response within < 15 min (Rank et al. 2016), BPDE triggered a rather moderate, but sustained PARP activation (> 8 h), potentially reflecting the kinetics and dynamics of the different DNA damage responses. We observed a PARP1-dependent decline in intracellular NAD^+^ pools after treatment with low micromolar concentrations of BPDE (Fig. 2), which is in line with results from Lin and Yang (Lin and Yang 2008). Interestingly, with low nanomolar concentrations of BPDE, we observed an increase in NAD^+^ levels 1 day after treatment, which was independent of PARP1 activity. The reasons for this PARP1-independent increase in NAD^+^ levels are unclear at the moment and need further evaluation. The PARP1-dependent decline of intracellular NAD^+^ is consistent with the finding that pharmacological PARP inhibition as well as genetic *PARP1* ablation led to slight, yet statistically significant, cytoprotective effects within the first 24 h after BPDE treatment (Fig. 3). In contrast, however, when analyzing the colony forming ability of HeLa cells after low nanomolar BPDE exposure 7 days after treatment, a strong PARP1-dependent sensitization effect towards BPDE was observed. This observation led to the initial assumption of an active role of PARP1 in the NER of BPDE-induced lesions, similar to what was reported for the role of PARP1 in the repair of UV-induced DNA lesions (Pines et al. 2012; Purohit et al. 2016; Robu et al. 2013, 2017; Vodenicharov et al. 2005). Using an HCRA, with which we previously showed a role for PARP activity in the repair of UV-induced DNA damage (Fischer et al. 2014), we now demonstrate that this assay can also be used to efficiently monitor the repair of BPDE-induced DNA lesions in extrachromosomal plasmid DNA (Suppl. Figure 6). However, in contrast to UV-induced damage, no influence of PARP activity was evident. This finding can potentially be explained by the fact that UV-induced and chemical-induced bulky adducts are not necessarily repaired in the same manner. Thus, e.g., the UV-damaged DNA-binding protein 2 (DDB2) is likely to participate in the removal of UV-DNA adducts, but not in the recognition of bulky adducts and crosslinks, because these lesions do not fit into the DDB2-binding pocket (Robu et al. 2017; Scrima et al. 2008). Interestingly, in particular, DDB2 has been shown to cooperate with PARP1 in the repair of UV-induced DNA damage (Pines et al. 2012; Robu et al. 2013), suggesting that the different outcomes on the role of PARylation in the repair of UV lesions and BPDE-DNA adducts indeed have a molecular basis.

The finding of a strong PARP1-dependent increase in 53BP1/yH2A.X foci after nanomolar treatment with BPDE (Fig. 7) points towards replication stress as an alternative NER-independent mechanism of how PARP1 participates in BPDE stress response. 53BP1/yH2A.X foci are indicative of DNA double-strand breaks, which can arise as a consequence of collapsed replication forks during replication stress (Gaillard et al. 2015; Panier and Boulton 2014; Rothkamm et al. 2015; Scully and Xie 2013). In general, a role for PARP1 in the replication stress response has been well established. Bryant et al. found that PARP1 binds to and becomes activated by stalled replication forks (Bryant et al. 2009), where it stabilizes the ‘chicken foot’ structure via inhibition of untimely RECQ1-mediated branch migration, thus providing time for DNA lesion removal before replication restart (Berti et al. 2013; Ray Chaudhuri et al. 2012). Furthermore, when stalled replication forks collapse and form one-ended DSBs, PARP1 facilitates HR-mediated repair and replication restart by recruitment of MRE11. PARP1 inhibition or knockout would in turn result in increased replication stress and a more dominant formation of DSBs and delayed repair of the latter (Berti et al. 2013; Bryant et al. 2009; Haince et al. 2008; Ray Chaudhuri et al. 2012).

To what extent BPDE can induce replication stress is not very well studied, but it would be plausible that BPDE-DNA adducts block fork progression during DNA synthesis. Consistently, a previous report showed that BPDE-DNA adducts are enriched at replication forks (Paules et al. 1988). In consequence, this could lead to fork stalling, collapsing and the formation of double-strand breaks (Gaillard et al. 2015). Results from the current study clearly reveal that BPDE treatment in nanomolar concentrations induces replication stress during S phase (Fig. 6). Furthermore, we provide strong evidence that PARP1 is a key factor to overcome BPDE-induced replication stress (Fig. 7). The initial induction of γH2A.X upon BPDE treatment was comparable between PARP1 proficient and deficient S-phase cells, implying induction of equal amounts of replication damage. However, over time (≥ 8 h), *PARP1* KO cells showed strongly increased levels of phosphorylated H2A.X compared to wild-type cells (Fig. 6a). Thus, it can be hypothesized that *PARP1* proficient cells readily counteract the induced replication stress, prevented strand breakage, or efficiently repaired collapsed forks. *PARP1* KO cells, however, accumulated more collapsed replication forks and DSBs, as evident by the strong increase of 53BP1/yH2A.X, and were less capable to timely repair these by HR. Such a model is supported by the long-lasting low-level increase in PAR formation over several hours, since in such unsynchronized cultures, cells enter S phase at different timepoints, leading to constant replication stress in the cell population.

In general, blocked replication forks can be handled in two ways. The damage is either repaired by homologous recombination or bypassed by translesion synthesis polymerases (Gaillard et al. 2015). Both error-free as well as error-prone translesion bypass have been described (Christmann et al. 2016; Li et al. 2002; Temviriyanukul et al. 2012), the latter of which was shown to be at least in part responsible for overall BPDE mutagenicity. Thus, it is well conceivable that the potentiation of BPDE mutagenicity after PARP inhibition (Fig. 8) can be explained by a shift from homologous recombination repair to translesion synthesis. Considering the findings from the current study, it is tempting to speculate that an impaired PARylation response may contribute to an increased risk for B[a]P-induced mutations and tumor formation on the organismic level, e.g., in cigarette smoke-induced lung tumors (Li et al. 2017).

## Electronic supplementary material

Below is the link to the electronic supplementary material.

Supplementary material 1 (PDF 655 KB)
