# Supplementary material for: PARP1 protects from benzo[a]pyrene diol epoxide-induced replication stress and mutagenicity
Source: Arch Toxicol. 2017 Dec 1;92(3):1323–40. doi: 10.1007/s00204-017-2115-6 (PMC5866831; doi:10.1007/s00204-017-2115-6)
Supplement: Supplementary file 1 — Supplementary material 1 (PDF 655 KB) [file 204_2017_2115_MOESM1_ESM.pdf]

Supplementary information

PARP1 protects from benzo[a]pyrene diol epoxide-induced replication stress and mutagenicity

Jan MF Fischer[§,1,2], Tabea Zubel[§,1,2], Kirsten Jander[1], Jelena Fix[1], Irmela Trussina[1], Daniel Gebhard[3], Jörg Bergemann[3], Alexander Bürkle[1,*], and Aswin Mangerich[1,*]

[§]Shared first authorship

[1]Molecular Toxicology Group, Department of Biology, University of Konstanz, Germany

[2]Konstanz Research School Chemical Biology, University of Konstanz, Germany

[3]Department of Life Sciences, Albstadt-Sigmaringen University of Applied Sciences, Germany

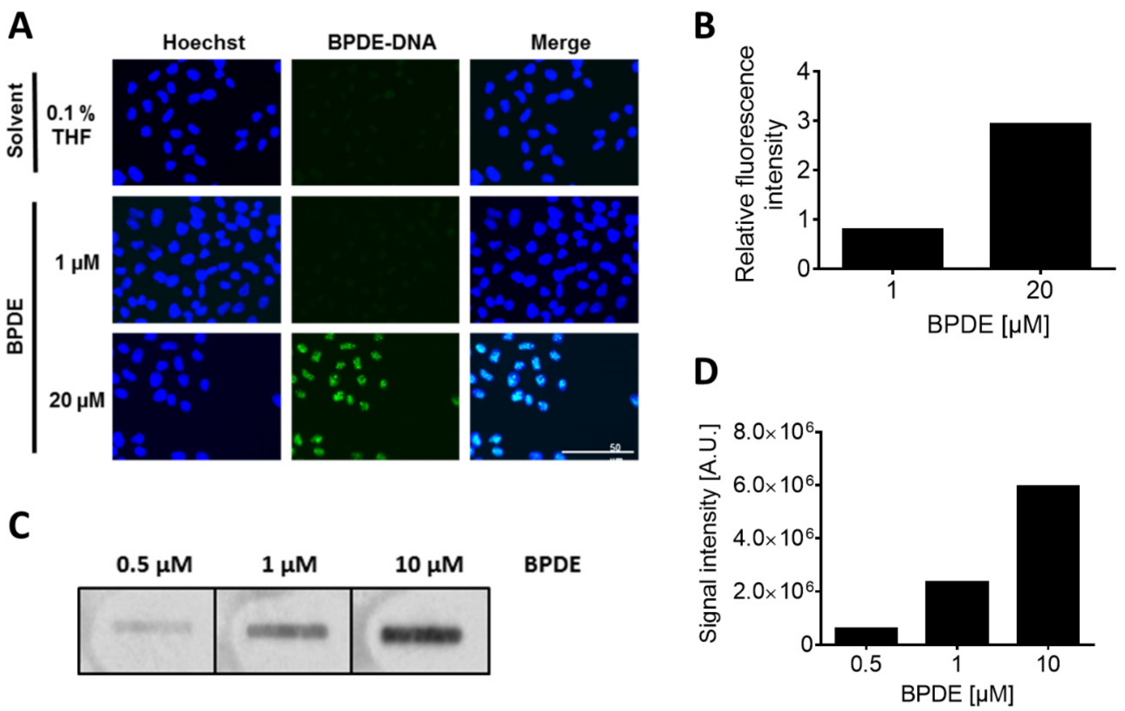

Suppl. Fig. 1. Immunochemical analysis of BPDE-induced DNA adducts in HeLa cells. A. Representative immunofluorescence images of BPDE-DNA adduct staining. Cells were treated with BPDE in concentrations as indicated for 1 h and DNA lesions were detected with a BPDE-DNA specific antibody. **B.** Densitometric quantification of **A**. Depicted are the means of signal intensities of >100 cells, normalized to solvent control. **C.** Slot blot analysis. Cells were treated with BPDE in concentrations as indicated for 1 h. Cellular DNA was extracted, blotted on a nylon membrane and DNA lesions were detected with a BPDE-DNA specific antibody. **D.** Densitometric quantification of the chemiluminescence signal of **C**.

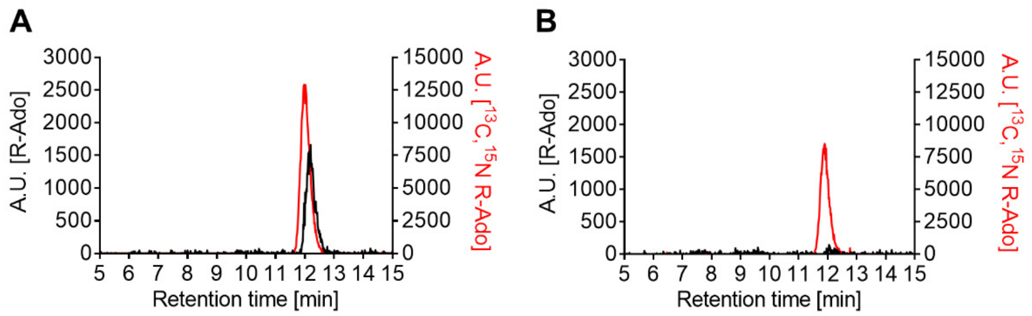

Suppl. Fig. 2. Representative LC-MS/MS chromatograms. HeLa cells were treated with 50 µM **BPDE for 1 h. A.** Chromatogram of ABT888-untreated cells. **B.** Representative chromatogram of ABT888-treated cells.

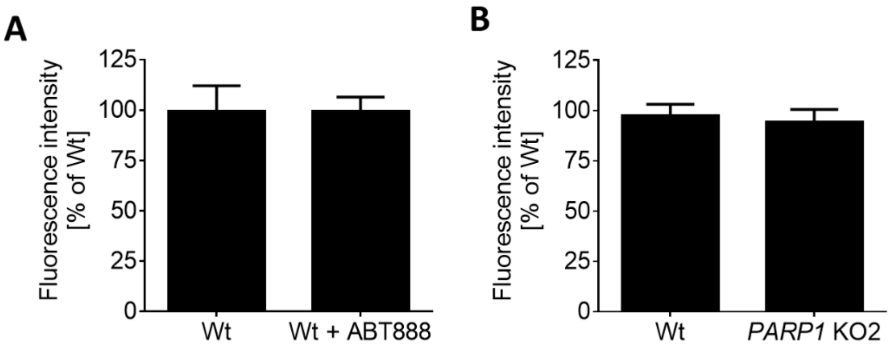

Suppl. Fig. 3. Solvent controls of the Alamar Blue assay. PARP inhibition (**A**) or genetic ablation (**B**) of *PARP1* had no influence on cell proliferation and metabolism (n=4). Data represent means ± SEM of ≥3 independent experiments, each performed in ≥3 technical replicates.

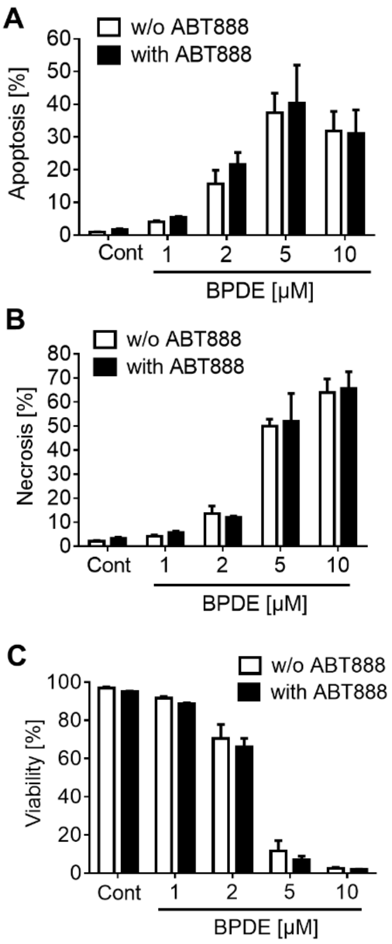

Suppl. Fig. 4. PARP inhibition does not affect BPDE-induced cell death. A-C. HeLa cells were treated with increasing concentrations of BPDE. 48 h after exposure, the viability as well as early apoptotic and late apoptotic / necrotic portion of cells was detected by Annexin V / PI staining and flow cytometric analysis. While for BPDE a dose-dependent induction of apoptosis (**B**) and necrosis (**C**) could be observed, PARP inhibition had no detectable influence on cell death at the time point analyzed. **A-D.** Data represent means ± SEM of three independent experiments. Statistical evaluation was performed using 2-way ANOVA analysis followed by Sidak's multiple comparison test.

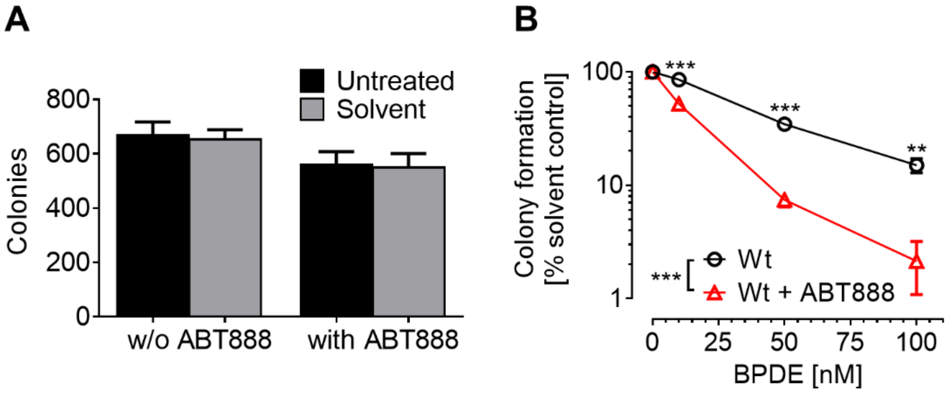

Suppl. Fig. 5. Supplementary data on clonogenic survival assay. A. Quantification of control assays, *i.e.*, solvent control or treated with ABT888. While the presence of solvent (THF/TEA) did not influence colony formation, a weak, yet not significant, decrease was caused by ABT888. **B.** Quantification of **Fig. 4 A**. HeLa cells were treated with increasing concentrations of BPDE for 30 min and clonogenic survival assays were performed. Data represent means ± SEM of three independent experiments, each performed in technical triplicates. Statistical evaluation was performed using 2-way ANOVA analysis followed by Sidak's multiple comparison testing.

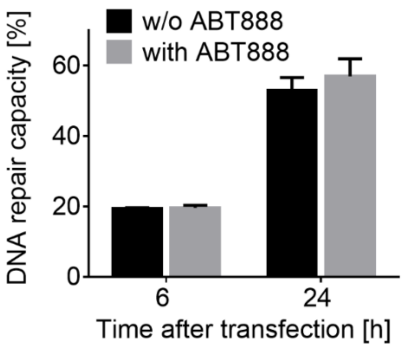

Suppl. Fig. 6. PARP inhibition has no impact on the direct repair of BPDE-DNA adducts. A host cell reactivation assay was performed with a BPDE-treated (75 µM) reporter plasmid. After 6 and 24 h the functional reconstitution of plasmids was assessed by analyzing the numbers of cells with reporter gene expression (*dsRed*). PARP inhibition did not alter the cellular NER capacity. Data represent means ± SEM of three independent experiments. Statistical evaluation was performed using 2-way ANOVA analysis followed by Sidak's multiple comparison test.

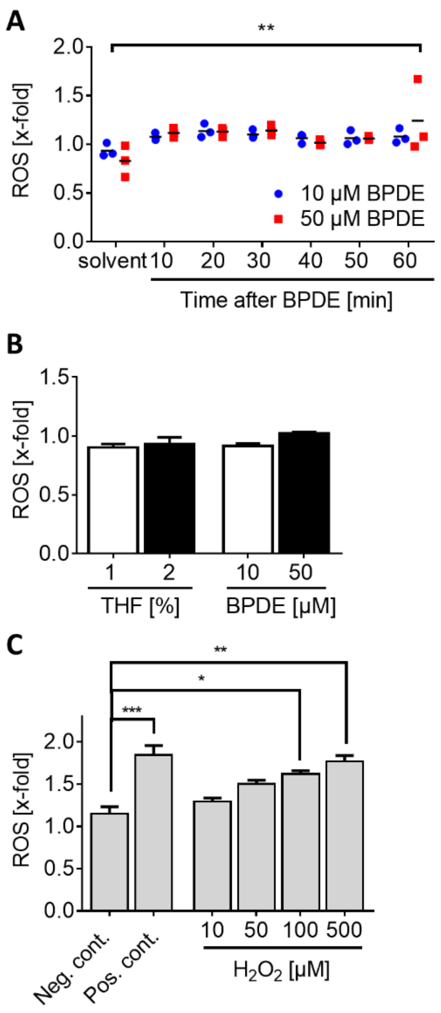

Suppl. Fig. 7. Analysis of ROS formation upon BPDE treatment. A-B. Dihydroethidium (DHE) was used after BPDE treatment as an indicator for ROS formation. **A.** Cells were treated with BPDE (10 or 50 µM) and after the indicated time points (≤ 1 h) DHE was added and fluorescence was detected. Using a high dose of 50 µM BPDE, a weak increase in ROS formation could be observed within the first hour after BPDE treatment. **B.** Longer exposure (5 h) to BPDE did not result in elevated levels of ROS formation. **C.** Control treatments for ROS formation (n=3). HeLa Kyoto cells were treated first with the antioxidant N-acetyl cysteine (NAC) and subsequently with the ROS inducer tert-butyl hydroperoxide (TBHP, neg. control) or with TBHP only (pos. control). A dose-dependent increase in ROS levels could be observed with H_2O_2. **A-C.** Data represent means ± SEM of three independent experiments, each performed in technical triplicates, normalized to untreated control. Statistical evaluation was performed using 2-way ANOVA analysis followed by Sidak's multiple comparison test. ** p>0.01

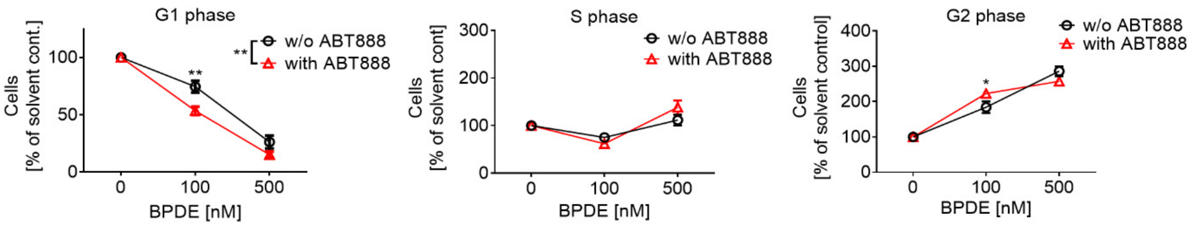

Suppl. Fig. 8. PARP inhibition influences the BPDE-induced cell cycle delay in unsynchronized HeLa cells. A. Cells were incubated with 10 µM ABT888 before and after treatment with increasing concentrations of BPDE for 1 h. Thereafter, cells were cultured for 24 h and cell cycle phase distribution was determined by using PI and analyzing the DNA content. At 100 nM BPDE significant fewer cells were found in G1 phase after 24 h when PARP activity was inhibited. PARP inhibition did not influence the numbers of S phase cells in response to BPDE treatment, but ABT888 treated cells further accumulated in G2 phase of the cell cycle. Data represent means ± SEM of ≥3 independent experiments, normalized to solvent control. Statistical evaluation was performed using 2-way ANOVA analysis followed by Sidak's multiple comparison test. * $p<0.05$, ** $p<0.01$.
